# *Ehrlichia chaffeensis* Uses Its Surface Protein EtpE to Bind GPI-Anchored Protein DNase X and Trigger Entry into Mammalian Cells

**DOI:** 10.1371/journal.ppat.1003666

**Published:** 2013-10-03

**Authors:** Dipu Mohan Kumar, Mamoru Yamaguchi, Koshiro Miura, Mingqun Lin, Marek Los, Johannes F. Coy, Yasuko Rikihisa

**Affiliations:** 1 Department of Veterinary Biosciences, College of Veterinary Medicine, The Ohio State University, Columbus, Ohio, United States of America; 2 Department of Clinical & Experimental Medicine, Integrative Regenerative Medical Center Linköping University, Linkoping, Sweden; 3 Vorstand/CSO TAVARLIN AG, Darmstadt, Germany; Northwestern University Feinberg School of Medicine, United States of America

## Abstract

*Ehrlichia chaffeensis,* an obligatory intracellular rickettsial pathogen, enters and replicates in monocytes/macrophages and several non-phagocytic cells. *E. chaffeensis* entry into mammalian cells is essential not only for causing the emerging zoonosis, human monocytic ehrlichiosis, but also for its survival. It remains unclear if *E. chaffeensis* has evolved a specific surface protein that functions as an ‘invasin’ to mediate its entry. We report a novel entry triggering protein of *Ehrlichia,* EtpE that functions as an invasin. EtpE is an outer membrane protein and an antibody against EtpE (the C-terminal fragment, EtpE-C) greatly inhibited *E. chaffeensis* binding, entry and infection of both phagocytes and non-phagocytes. EtpE-C-immunization of mice significantly inhibited *E. chaffeensis* infection. EtpE-C-coated latex beads, used to investigate whether EtpE-C can mediate cell invasion, entered both phagocytes and non-phagocytes and the entry was blocked by compounds that block *E. chaffeensis* entry. None of these compounds blocked uptake of non-coated beads by phagocytes. Yeast two-hybrid screening revealed that DNase X, a glycosylphosphatidyl inositol-anchored mammalian cell-surface protein binds EtpE-C. This was confirmed by far-Western blotting, affinity pull-down, co-immunoprecipitation, immunofluorescence labeling, and live-cell image analysis. EtpE-C-coated beads entered bone marrow-derived macrophages (BMDMs) from wild-type mice, whereas they neither bound nor entered BMDMs from DNase X^-/-^ mice. Antibody against DNase X or DNase X knock-down by small interfering RNA impaired *E. chaffeensis* binding, entry, and infection. *E. chaffeensis* entry and infection rates of BMDMs from DNase X^-/-^ mice and bacterial load in the peripheral blood in experimentally infected DNase X^-/-^ mice, were significantly lower than those from wild-type mice. Thus this obligatory intracellular pathogen evolved a unique protein EtpE that binds DNase X to enter and infect eukaryotic cells. This study is the first to demonstrate the invasin and its mammalian receptor, and their *in vivo* relevance in any ehrlichial species.

## Author Summary

Human monocytic ehrlichiosis (HME), discovered in 1986, was designated as a nationally notifiable disease by Centers for Disease Control and Prevention in 1998. HME is one of the most prevalent, life-threatening emerging infectious diseases in the United States. HME is caused by a bacterium, *Ehrlichia chaffeensis* and is transmitted by the bite of infected ticks. This bacterium has special ability to enter and replicate inside human white blood cells and this feature is very essential for the bacterial survival. How *E. chaffeensis* enters host cells has been a mystery. The present study revealed that *E. chaffeensis* outer-surface protein named EtpE binds a specific host cell-surface protein, DNase X, and this ligand-receptor interaction is required to induce bacterial entry into its host cells. In order to test whether *E. chaffeensis* infection can be prevented by EtpE immunization, mice were immunized with the recombinant EtpE protein, and challenged with live *E. chaffeensis*. Infection was significantly reduced in the EtpE protein-immunized mice compared to controls. Mice lacking DNase X were also resistant to infection. This study shows EtpE-mediated entry pathway of *E. chaffeensis* is important in infecting mammals and EtpE can be incorporated into a future HME vaccine design.

## Introduction

*Ehrlichia chaffeensis* causes human monocytic ehrlichiosis (HME), an emerging tick-borne zoonosis. From the site of infected tick bite on human skin, *E. chaffeensis* infects monocytes and spreads via the bloodstream to various tissues, causing a systemic febrile disease. HME is characterized by fever, headache, myalgia, thrombocytopenia, leucopenia, and elevated liver-enzyme levels, but complications such as pulmonary insufficiency, renal failure, encephalopathy, and disseminated intravascular coagulation can cause death [1]. Early diagnosis and the proper treatment with doxycycline are critical to prevent complications. The disease is of particular threat to the immunocompromised and the elderly people [1].

*E. chaffeensis* is a small obligatory intracellular bacterium. It belongs to the family Anaplasmataceae in the order Rickettsiales that includes many understudied pathogens of veterinary and public health importance [2]. By electron microscopy, *E. chaffeensis* is a polymorphic bacterium (0.2–2.0 µm in diameter), and can be morphologically categorized as small dense-cored cells (DCs) or large reticulate cells (RCs) [3]. DCs are ∼0.2–0.5 µm in diameter, which is close to the size of the elementary body of *Chlamydia* and larger viruses such as *Vaccinia* virus. By light microscopy, it is not possible to distinguish individual RCs and DCs, since *E. chaffeensis* aggregates inside eukaryotic host cells. The characteristic clump of intracellular *E. chaffeensis* organisms is termed as “morula” (mulberry in Latin) [2]. However, when they are freshly isolated from host cells and dispersed, smaller bacteria (<0.5 µm) are more densely stained with basic dye than larger bacteria (>0.5 µm); therefore, they were defined as DCs and RCs, respectively [4]. DCs are more resistant to strong sonication and more infectious than RCs [5]. In cell culture, a biphasic developmental cycle has been reported: initially small infectious DCs bind to and internalize into host cells, which then develop into larger replicating RCs inside a membrane-lined compartment that resembles early endosomes. After replication in expanding inclusions, the mature RCs transform back into DCs prior to release from the host cells [4], [5]. In patients' blood specimens, monocytes were primarily infected with *E. chaffeensis*, and hence, the disease was named as “monocytic ehrlichiosis” to distinguish it from “granulocytic ehrlichiosis” caused by infection with granulocyte-tropic *Ehrlichia* sp. [1]. *E. chaffeensis* can replicate well in several mammalian cell lines including canine histiocytic leukemia (DH82), human acute leukemia (THP-1), human promyelocytic leukemia (HL-60), human embryonic kidney (HEK293), and monkey endothelial (RF/6A) cells [6]–[8].

Entry into the permissive eukaryotic host cells is essential for *E. chaffeensis* to sustain its life, since its small genome of 1.18 Mb lacks a large portion of metabolic genes that are required for free living [9]. *E. chaffeensis* also lacks LPS, peptidoglycan, lipoteichoic acid, and flagella that engage Toll-like or NOD-like receptors, or scavenger receptors [2], [10]. *E. chaffeensis* entry and subsequent infection of THP-1 cells, but not binding are almost completely inhibited by monodansylcadaverine (MDC), a transglutaminase inhibitor [11]. MDC is known to block *Neorickettsia risticii* (formerly *Ehrlichia risticii*) entry and infection of P388D_1_ cells, vesicular stomatitis virus uptake and receptor-mediated endocytosis of α2-macroglobulin by Swiss 3T3 mouse cells, but not the uptake of latex beads by P388D_1_ mouse macrophages [12]–[14]. *E. chaffeensis* entry into THP-1 cells, leading to productive infection, is dependent on the host-cell surface lipid rafts and glycosylphosphatidyl inositol (GPI)-anchored proteins [15]. Furthermore, lipid raft-associated protein caveolin-1, but not clathrin localizes to the *E. chaffeensis* entry site [15]. After entry, *E. chaffeensis* replicates in the membrane-bound compartment resembling an early endosome as it contains early endosome antigen 1 (EEA1), Rab5, and transferrin receptor [6]. Several intracellular bacteria are known to enter host cells by using their specific surface protein collectively called as ‘invasin’ or ‘internalin’ [16]. However, detailed mechanisms of *E. chaffeensis* entry are unknown; particularly regarding the involvement of any specific bacterial surface protein that can function as an invasin and its cognitive host-cell receptor [2].

The comparative genome hybridization study of *E. chaffeensis* strains revealed that a hypothetical protein, ECH1038, consists of highly conserved N- and C-terminal segments flanking its strain-variable central region [17]. ECH1038 expression is up-regulated in the DC stage of *E. chaffeensis* [18]. In this study, we uncovered that ECH1038 (here named as entry triggering protein of *Ehrlichia*, EtpE), particularly its C-terminal conserved region (EtpE-C), is critical for *E. chaffeensis* binding, entry, and infection of several different host cell types. In order to study whether EtpE-C can mediate the invasion into permissive host cells in the absence of other *E. chaffeensis* factors, we used latex beads coated with EtpE-C. The coated beads entered both phagocytes and non-phagocytes in a manner similar to *E. chaffeensis* entry. Subsequent investigation led to the discovery that DNase X, a host cell surface GPI-anchored protein, is the receptor of EtpE-C mediating the entry of *E. chaffeensis* into several mammalian cell types permissive to its replication.

## Results

### ECH1038 (EtpE) is highly expressed by *E. chaffeensis* in mammalian cells

ECH1038 (GenBank accession no. YP_507823, EtpE) consists of 1963 amino acid residues (M_r_ 222,638, pI: 7.0) and is predicted to be an outer membrane protein with an N-terminal secretion signal by PSORT analysis [17]. Although EtpE is variable in the central ∼950 residues, the N-terminal ∼700 residues and C-terminal ∼300 residues are conserved among multiple *E. chaffeensis* strains of distinct virulence, suggesting that these two regions are indispensable for *E. chaffeensis*. The amino acid sequence alignment of EtpE orthologs in the three strains of *E. chaffeensis*, Arkansas, Wakulla, and Liberty are shown (suppl. Fig. S1). Amino acid sequence alignment of EtpE orthologs among three genome-sequenced *Ehrlichia* species, *E. chaffeensis* Arkansas, *E. canis* Jake and *E. ruminantium* Welgevonden, revealed that while most of the N-terminal proximal region was conserved also among *Ehrlichia* species, the C-terminal region was not (suppl. Fig. S2).

**Figure 1:**
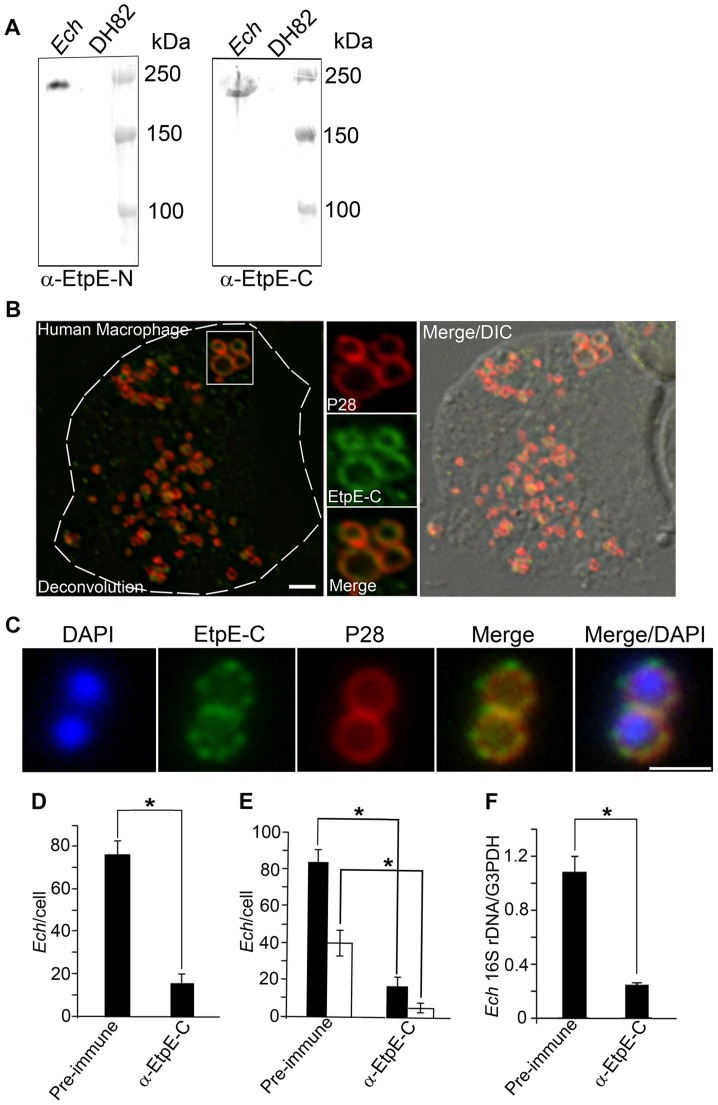
EtpE-C is exposed at the bacterial surface, and anti-EtpE-C neutralizes *E. chaffeensis* infection *in vitro*. (A) Western blot analysis of *E. chaffeensis-*infected (*Ech*) and uninfected DH82 cells at 60 h pi using anti-EtpE-N (α-EtpE-N) and anti-EtpE-C (α-EtpE-C). (B) Double immunofluorescence labeling of *E. chaffeensis-*infected human primary macrophages derived from peripheral blood monocytes at 56 h pi. Cells were fixed with PFA, permeabilized with saponin, and labeled with anti-EtpE-C and anti-*E. chaffeensis* major outer membrane protein P28. The white dashed line denotes the macrophage contour. The boxed region indicates the area enlarged in the smaller panels to the right. Merge/DIC: Fluorescence images merged with Differential interference contrast image (DIC). A single *z*-plane (0.4 µm thickness) by deconvolution microscopy was shown. Scale bar, 2 µm. (C) *E. chaffeensis* was incubated with DH82 cells for 30 min and double immunofluorescence labeling was performed using anti-EtpE-C and anti-*E. chaffeensis* P28 without permeabilization. DAPI was used to label DNA. Scale bar, 1 µm (see also suppl. Fig. S2). (D) Numbers of *E. chaffeensis* bound to RF/6A cells at 30 min pi. Host cell-free *E. chaffeensis* was pretreated with anti-EtpE-C or preimmune mouse serum and incubated with RF/6A cells for 30 min. Unbound *E. chaffeensis* was washed away, cells were fixed with PFA, and *E. chaffeensis* labeled with anti-P28 without permeabilization. *E. chaffeensis* in 100 cells were scored. (E) Numbers of *E. chaffeensis* internalized into RF/6A cells at 2 h pi. *E. chaffeensis* was pretreated with anti-rEtpE-C or preimmune mouse serum and incubated with RF/6A cells for 2 h. To distinguish intracellular from bound *E. chaffeensis*, unbound *E. chaffeensis* was washed away and cells were processed for two rounds of immunostaining with anti-P28; first without permeabilization to detect bound but not internalized *E. chaffeensis* (AF555–conjugated secondary antibody) and second round with saponin permeabilization to detect total *E. chaffeensis*, i.e., bound plus internalized (AF488–conjugated secondary antibody). *E. chaffeensis* in 100 cells was scored. The black bar represents total *E. chaffeensis* and the white bar represents internalized *E. chaffeensis* (total minus bound) (see also suppl. Fig. S3). (F) Infection of RF/6A cells with *E. chaffeensis* at 48 h pi. *E. chaffeensis* was pretreated with anti-EtpE-C or preimmune mouse serum and used to infect RF/6A cells; cells were harvested at 48 h pi. qPCR for *E. chaffeensis* 16S rDNA was normalized with G3PDH DNA. Data represent the mean and standard deviation of triplicate samples and are representative of three independent experiments. *Significantly different (*P*<0.05).

To begin probing EtpE function, we cloned N-terminal (residues 29–708) and C-terminal (residues 1658–1965) of EtpE as C-terminal histidine-tagged fusion proteins (rEtpE-N and rEtpE-C) and antisera were prepared against rEtpE-N and rEtpE-C. Western blot analysis using these antibodies showed that full-length EtpE was expressed by *E. chaffeensis* in DH82 cells (Fig. 1A). DH82 cells were initially used, since this cell line has been successfully used to culture isolate *E. chaffeensis* from the blood of HME patients [19], [20].

To determine whether EtpE is expressed by *E. chaffeensis* in human monocytes, the pathogen's primary *in vivo* target cells, EtpE expression was determined in *E. chaffeensis* cultured in human primary macrophages derived from peripheral blood monocytes by double immunofluorescence labeling after paraformaldehyde (PFA) fixation and saponin permeabilization. *E. chaffeensis* major outer membrane protein P28 [21] was used as positive control to label the bacterial membrane. The results showed that EtpE was abundantly expressed by *E. chaffeensis* in human macrophages, and localized at bacterial membrane like P28 [21] (Fig. 1B).

### EtpE is exposed on the *E. chaffeensis* surface, and anti-EtpE-C inhibits *E. chaffeensis* binding, entry, and infection

P28 is bacterial surface exposed [21] and is a β-barrel protein that functions as porin [22]. To determine whether EtpE is exposed on the bacterial surface, double immunofluorescence labeling with anti-EtpE-C and anti-P28 was performed after PFA fixation without saponin permeabilization using *E. chaffeensis* bound to the surface of DH82 cells. Unlike methanol or acetone fixation, PFA fixation does not allow antibody penetration across biological membranes unless with subsequent permeabilization, thereby limiting antibody staining to molecules exposed to the cell surface [23]. The result showed labeling of both EtpE and P28 (Fig. 1C). Of note, labeled EtpE on *E. chaffeensis* had a beaded (rosary-like) pattern encircling individual bacterium, in contrast to P28 that had a uniform ring pattern (Fig. 1C). When host cell-free bacteria were treated with pronase E, the surface immunofluorescence staining of EtpE was abolished, but not of CtrA which is an *E. chaffeensis* cytosolic response regulator of a two-component system [18] (suppl. Fig. S3). These data indicate the surface exposure of EtpE. In contrast to the punctate labeling pattern of EtpE in host cell-bound bacteria, homogeneous labeling of EtpE was observed on host cell-free bacteria (suppl. Fig. S3).

Given the surface exposure of EtpE on *E. chaffeensis*, we examined whether the antibody against EtpE inhibits binding, entry, and infection of *E. chaffeensis*. Among the several host cell types used in this study, primary monocytes, macrophages, and myelocytic leukemia cell lines (DH82 and THP-1 cells) are referred to as phagocytes. Phagocytes are very efficient in bacteria and particle uptake as they have an array of dedicated phagocytic receptors, including pathogen pattern recognition receptors, mannose receptors, scavenger receptors, receptors for immunoglobulin (FcR) and complement (CR3) that utilize opsonins for ingestion, to name a few [10], [24]. The other two cell lines used in this study, RF/6A endothelial and HEK293 epithelial cells, are referred to as non-phagocytes, since they lack these features. We first used non-phagocytes to study the effect of *in vitro* antibody neutralization of EtpE as they lack the response to opsonization and will not readily take-up opsonized particles. *E. chaffeensis* was pre-incubated with mouse anti-EtpE-C or preimmune mouse sera, and then incubated with RF/6A cells. Binding and entry were determined by immunofluorescence labeling of *E. chaffeensis* with anti-P28 at 30 min and 2 h post-incubation/infection (pi), respectively. Infection was determined at 48 h pi by quantitative real-time PCR (qPCR). Anti-EtpE-C blocked *E. chaffeensis* binding, entry, and subsequently infection by approximately 80% compared to preimmune serum (Fig. 1, D–F). Similar level of inhibition of binding and entry was observed using mouse anti-EtpE-C in phagocytic cells such as human THP-1 cells (suppl. Figs. S4A and S4B) and canine DH82 cells (data not shown). This suggests that human or canine FcR-mediated entry of *E. chaffeensis* opsonized with mouse anti-EtpE-C was negligible in this experiment. Immunization of mice with recombinant P28, which functions as a porin [22], protects mice from *E. chaffeensis* challenge [21]. Additionally, in a mouse model of HME, immunization of mice with *Ehrlichia muris* P28 conferred protection from *E. muris* challenge [25]. As another control, to rule out the possibility that inhibition of binding is a general property of antibody neutralization of any *E. chaffeensis* cell surface proteins, we examined whether antibody against P28 blocks *E. chaffeensis* binding and entry. Our result showed antibody (Fab fragment) against P28 did not block binding or entry of *E. chaffeensis* (suppl. Fig. S5A and S5B). Taken together, these results suggest that EtpE-C potentially serves as an invasin to trigger *E. chaffeensis* entry in both phagocytes and non-phagocytes.

EtpE is predicted to be anchored on the bacterial outer membrane at its N-terminus, based on analysis using the PRED-TMBB webserver [17], [26]. In contrast to anti-EtpE-C, anti-EtpE-N-pretreatment reduced *E. chaffeensis* infection by only 20% (suppl. Fig. S6A). Since both anti-EtpE-N and anti-EtpE-C reacted with native EtpE protein from *E. chaffeensis* equally well by Western blot analysis, we tested accessibility of anti-EtpE-N to EtpE molecules on live *E. chaffeensis* surface. For this purpose, we freshly prepared the host cell-free *E. chaffeensis* and incubated with the antibodies without pre-fixation. The result showed that *E. chaffeensis* was not as readily labeled with anti-EtpE-N as with anti-EtpE-C (suppl. Fig. S6B), suggesting that the antibody access to the N-terminal conserved region might be limited in the native conformation of EtpE in live *E. chaffeensis*.

### EtpE is expressed in HME patients and infected dogs, and immunization with rEtpE-C suppresses *E. chaffeensis* infection in mice

**Figure 2:**
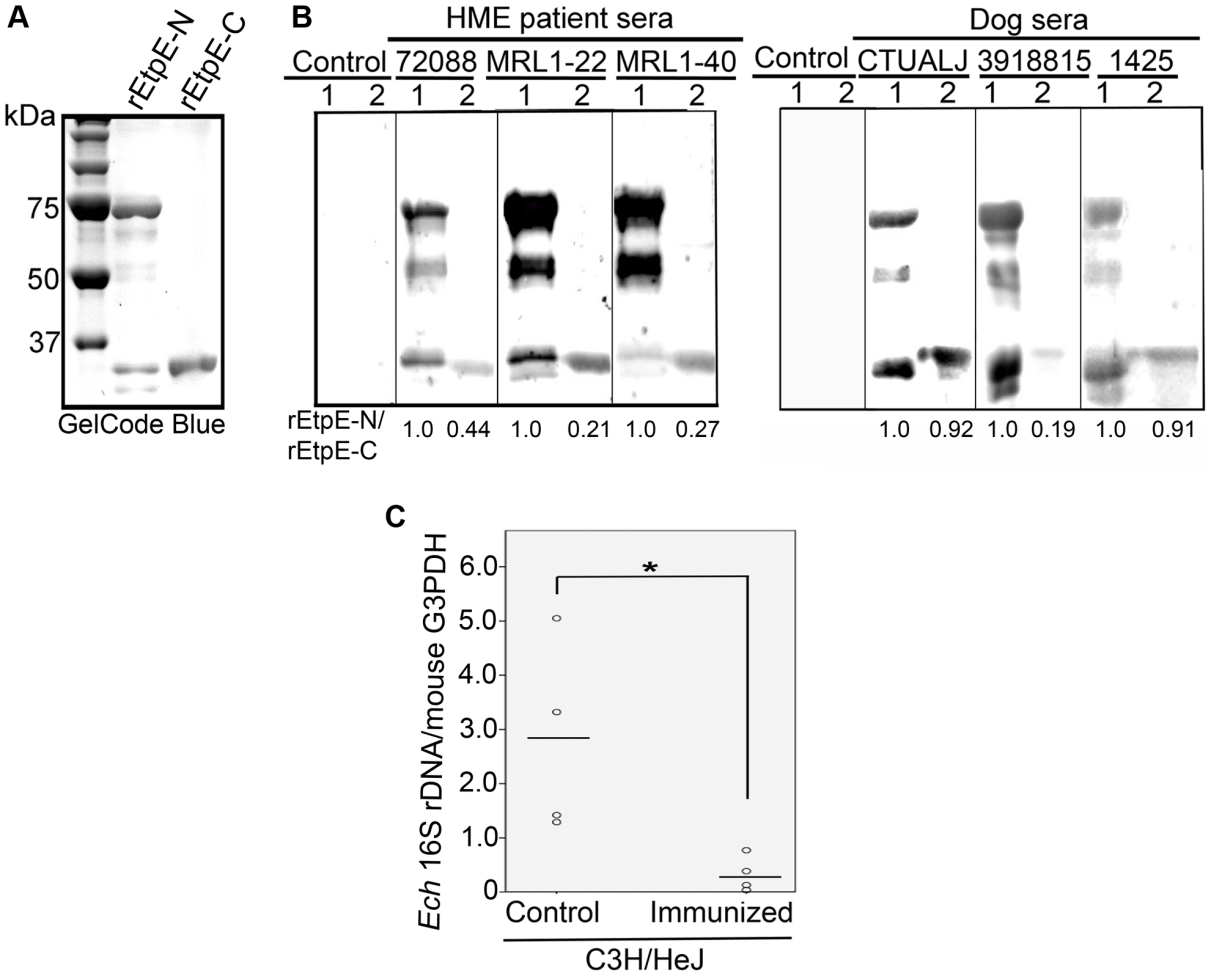
EtpE is expressed by *E. chaffeensis* in HME patients and infected dogs, and immunization with rEtpE-C protects mice against *E. chaffeensis* challenge. (A) SDS-PAGE analysis and GelCode Blue staining of rEtpE-N (lane 1) and rEtpE-C (lane 2) (5 µg/lane). rEtpE-N was partially cleaved after its expression in *E. coli* and thus is visualized as multiple bands. (B) Western blot analysis of rEtpE-N (lane 1) and rEtpE-C (lane 2) (5 µg/lane) with HME patient sera (ID: 72088, MRL1-22, MRL1-40) or control human serum (Control), or sera from dogs experimentally infected with *E. chaffeensis* (ID: CTUALJ, 3918815, 1425) or control dog serum. The relative band intensity for rEtpE-N/rEtpE-C (75 kDa and 34 kDa bands) assessed by densitometry was shown beneath the panels. (C) Dot-plot analysis of *E. chaffeensis* load of the blood samples from rEtpE-C-immunized and placebo-immunized mice at 5 days after *E. chaffeensis* challenge. qPCR of *E. chaffeensis* 16S rDNA normalized to mouse G3PDH DNA. *Significantly different (*P*<0.05).

Because EtpE is highly expressed by *E. chaffeensis* in mammalian cells *in vitro*, we next examined whether EtpE is expressed *in vivo* by Western blot analysis of defined HME patient sera [27]. Equal quantities of rEtpE-N and rEtpE-C (GelCode Blue staining shown in Fig. 2A) were used as antigens in the assay. Patient sera recognized both rEtpE-N and rEtpE-C, whereas the control serum from a healthy individual in an HME non-endemic region did not react with the recombinant proteins (Fig. 2B). Similarly, sera from dogs experimentally infected with *E. chaffeensis* [28], that were previously shown to recognize *E. chaffeensis* OmpA [18] and other *E. chaffeensis* lipoproteins [28], recognized both rEtpE-N and rEtpE-C, but the control dog serum did not (Fig. 2B). These data indicated that EtpE is expressed by *E. chaffeensis in vivo* during infection of its natural hosts, humans and dogs, and that an antibody (humoral) response is mounted against this protein during infection and disease.

Previous studies showed that antibodies contribute to immunity against *E. chaffeensis* in immunocompetent mice [29]. Given the facts that anti-EtpE-C neutralized *E. chaffeensis* binding, consequently entry and infection *in vitro*, EtpE was expressed by *E. chaffeensis in vivo* and that a humoral immune response was mounted in infected mammals, we decided to examine whether rEtpE-C immunization could confer protection in mice from *E. chaffeensis* challenge. C3H/HeJ strain of mice was used, since this strain was reported to serve as a useful model for studying *E. chaffeensis* infection [30]. At 10 days after the last immunization, all mice were challenged intraperitoneally with *E. chaffeensis*. The *E. chaffeensis* load in the blood from rEtpE-C-immunized mice at 5 days post challenge was significantly lower than that of non-immunized mice (Fig. 2C). These results indicate that rEtpE-C is a protective immunogen relevant in *E. chaffeensis* infection *in vivo*.

### Entry of rEtpE-C-coated beads into macrophages is blocked by compounds that block *E. chaffeensis* invasion

**Figure 3:**
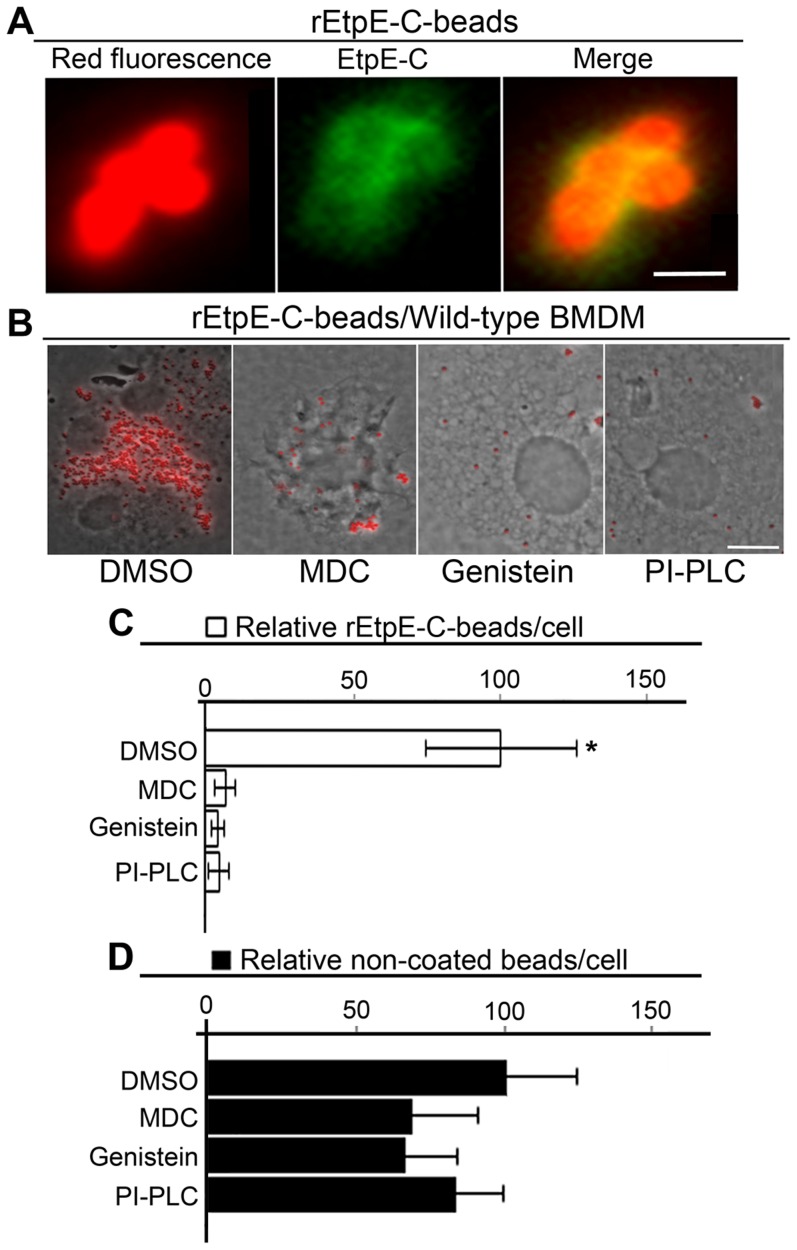
rEtpE-C-coated beads enter macrophages by a pathway similar to one that mediates *E. chaffeensis* entry. (A) Latex beads (red) coated with rEtpE-C by anti-EtpE-C labeling (green) under fluorescence microscopy. Scale bar, 1 µm. (B) Fluorescence and phase contrast merged images of rEtpE-C-coated beads incubated with mouse BMDMs. Cells were pretreated with DMSO (solvent control), MDC, genistein, or PI-PLC for 45 min followed by trypsin treatment to remove beads that were not internalized. Scale bar, 10 µm. (C and D) Numbers of internalized rEtpE-C-coated (C) and non-coated (D) beads/cell incubated with mouse BMDMs pretreated with MDC, genistein, or PI-PLC, relative to DMSO treatment (solvent control) set as 100. Data represent the mean and standard deviation of triplicate samples and are representative of three independent experiments. *Significantly different (*P*<0.05).

Bacterial surface exposure of EtpE-C and effectiveness of EtpE-C as the target for both *in vitro* and *in vivo* neutralization suggest that EtpE-C may mediate *E. chaffeensis* invasion. To investigate this possibility, we utilized fluorescent latex beads of average size of 0.5 µm (similar to the size of infectious DCs of *E. chaffeensis*) coated with rEtpE-C protein. The presence of rEtpE-C on beads was confirmed by dot-blot analysis (data not shown) and immunofluorescence labeling with antiserum against EtpE-C (Fig. 3A). Beads were incubated with mouse bone marrow-derived macrophages (BMDMs) for 45 min followed by trypsin treatment to remove beads that were not internalized. Mouse BMDMs were used here, also to serve as the wild-type control for the later studies using BMDMs from mutant mice. rEtpE-C-coated beads entered BMDMs (Figs. 3B and 3C). Treatment with MDC, genistein (broad-spectrum protein tyrosine kinase inhibitor), or phosphatidylinositol-specific phospholipase C (PI-PLC that removes GPI-anchored proteins from the cell surface) blocks *E. chaffeensis* entry and infection of THP-1 cells [11], [15]. The entry of rEtpE-C-coated beads into BMDMs was almost completely blocked by these treatments (Figs. 3B and 3C), suggesting rEtpE-C-coated beads enter BMDMs by the same signaling pathway as *E. chaffeensis* does. The latex bead is well-known to be taken up by macrophages and has been used as a model to study phagocytosis [31]. In striking contrast, entry of non-coated beads into BMDMs was not blocked by any of these treatments (Fig. 3D).

### rEtpE-C-coated beads enter non-phagocytes

**Figure 4:**
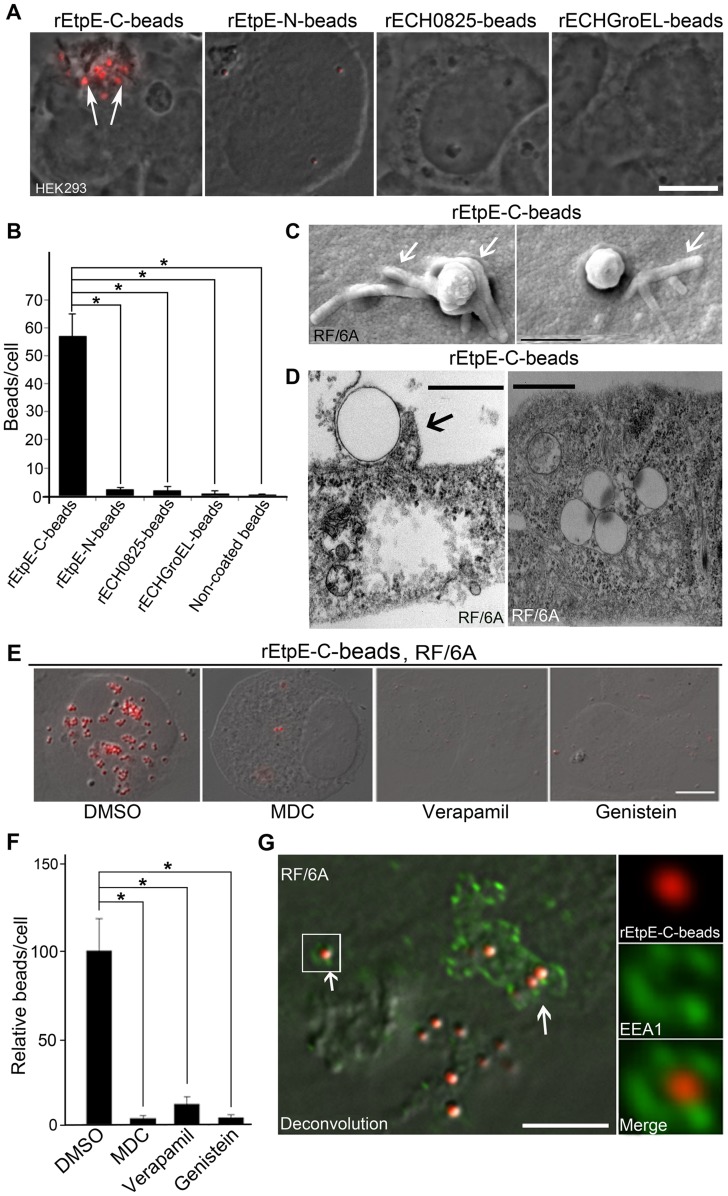
rEtpE-C-coated latex beads bind and enter non-phagocytic host cells. (A) rEtpE-C-coated beads (arrows), but not rEtpE-N, rECH0825, or rGroEL-coated beads, bind and enter HEK293 cells at 1 h pi. Scale bar, 10 µm. (B) Bar graph showing quantitation of similar experiment as (A) by scoring beads in 100 cells. Data represent the mean and standard deviation of triplicate samples and are representative of three independent experiments. *Significantly different (*P*<0.05). (C) Scanning electron micrograph of rEtpE-C-coated beads on the surface of RF/6A cells at 2 h pi. Note filopodia-like extensions embracing the beads (arrows). Scale bar, 1 µm. (D) Transmission electron micrograph of rEtpE-C-coated beads being engulfed (left panel) and internalized (right panel) into RF/6A cells at 8 h pi. Note filopodia-like extensions embracing the beads (arrow). Scale bars, 0.5 µm (left) and 1 µm (right). (E) Fluorescence and DIC images of rEtpE-C-coated beads in RF/6A cells. RF/6A cells were pretreated with DMSO (solvent), MDC, verapamil, or genistein for 30 min at 37°C, then incubated with rEtpE-C-coated beads for 8 h in the presence of compounds, washed and treated with trypsin to remove beads bound on the surface. A single *z*-plane (0.4 µm thickness) by deconvolution microscopy is shown here. Scale bar, 10 µm. (F) Bar graph showing numbers of internalized rEtpE-C-coated beads/cell of similar experiments as (E), relative to the number in DMSO treatment set as 100. Data represent the mean and standard deviation of triplicate samples and are representative of three independent experiments. *Significantly different (*P*<0.05). (G) Fluorescence and DIC merged images of RF/6A cells incubated with rEtpE-C-coated beads immunostained at 1 h pi with anti-EEA1 after permeabilization. Arrows indicate beads (red) surrounded with EEA1 (green). The boxed region is enlarged to the right. A single *z*-plane (0.2 µm thickness) by deconvolution microscopy is shown here. Scale bar, 5 µm.

Non-coated beads did not bind or enter RF/6A and HEK293 non-phagocytic cells (HEK293 cell data are shown in Fig 4B). Remarkably, rEtpE-C-coated beads did readily bind and enter non-phagocytes (HEK293 data are shown in Figs. 4A and 4B). Beads coated with other recombinant *E. chaffeensis* proteins including rEtpE-N, rECH0825 (a type IV secretion effector protein) [7] or rECH0365 (GroEL) did not bind HEK293 cells (Figs. 4A and 4B), indicating binding and entry of beads into non-phagocytes was due to specific coating with EtpE-C. Scanning and transmission electron microscopy revealed that rEtpE-C-coated beads bound to RF/6A cells were associated with filopodia-like membrane projections (Figs. 4C and 4D left panel) similar to those surrounding *E. chaffeensis* bound to DH82 cells [4]. Transmission electron microscopy of RF/6A cells incubated with rEtpE-C-coated beads verified that the beads were indeed internalized into RF/6A cells (Fig. 4D right panel). MDC, genistein, and verapamil (a Ca^2+^ channel blocker) that block *E. chaffeensis* entry into THP-1 cells [11], also blocked *E. chaffeensis* entry into RF/6A cells (suppl. Fig. S7, MDC data is shown). Treatment with any of these compounds almost completely blocked the entry of rEtpE-C-coated beads into RF/6A cells (Figs. 4E and 4F). Once internalized, *E. chaffeensis-*containing vacuoles acquire characteristics of early endosomes [6]. To determine whether rEtpE-C-coated beads were delivered to early endosomes, immunofluorescence labeling was used to visualize the spatial relationship of the early endosomal marker, EEA1 with the rEtpE-C-coated beads, and observed by deconvolution microscopy. Individual as well as multiple beads were seen encased by EEA1-labeled membranous compartment, suggesting that some beads were in endosomes (Fig. 4G). These results indicate that EtpE-C is an invasin, and even in the absence of any other *E. chaffeensis* factors, EtpE-C alone is sufficient to mediate the binding and entry of EtpE-C-coated beads into non-phagocytic cells.

### EtpE-C binds DNase X

Since EtpE-C could mediate binding and entry of EtpE-C-coated beads, we next searched for the potential host-cell receptor for EtpE-C. We cloned EtpE-C into the yeast two-hybrid bait vector and screened a human bone marrow cDNA prey library to identify interacting proteins. Of the 5 clones detected and sequenced, all of them encoded a protein, deoxyribonuclease 1-like 1 (DNase 1L1, DN1L1, or DNase X on chromosome Xq28, GenBank accession no: X90392, 302 residues). One of the clones contained an additional plasmid encoding S-adenosyl methionine-dependent methyltransferase but the coding sequence was out-of-frame; this prey construct alone did not support yeast growth when co-transformed with bait vector to test their interaction. All sequence hits corresponded to the C-terminal fragment of DNase X (residues 105–302). DNase X, one of the human DNase I–family endonucleases, is expressed on the cell surface as a GPI-anchored protein and also localized at early endocytic vesicles, endoplasmic reticulum, and Golgi [32], [33].

**Figure 5:**
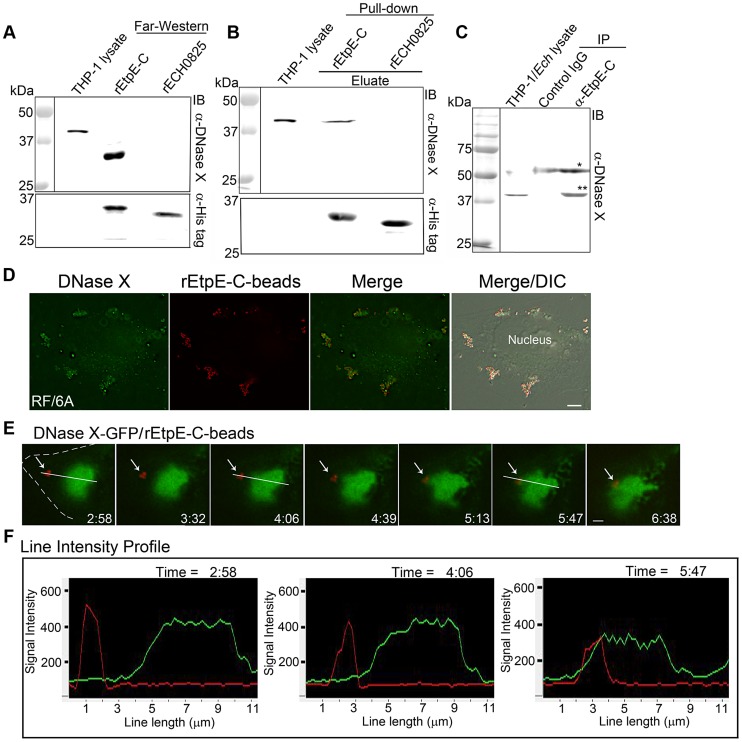
EtpE-C binds DNase X. (A) Far-Western blotting of renatured rEtpE-C and rECH0825 on a nitrocellulose membrane incubated with THP-1 cell lysate. Native DNase X was detected with anti-DNase X (α-DNase X), and recombinant proteins were detected with anti-histidine-tag (α-His tag). (B) Western blotting of THP-1 cell lysate following affinity pull-down with rEtpE-C bound to Ni-silica matrix. Bound proteins were eluted with imidazole and labeled with α-DNase X or α-His tag. (C) Western blot analysis of *E. chaffeensis-*infected THP-1 cell lysate immunoprecipitated with anti-EtpE-C (α-EtpE-C) or control IgG. THP-1 cells were incubated with *E. chaffeensis* for 30 min, followed by lysis, and immunoprecipitated with α-EtpE-C- or control mouse IgG-bound protein A agarose. The precipitates were subjected to Western blotting with α-DNase X. ** DNase X, * mouse IgG heavy chain. (D) Immunofluorescence labeling of rEtpE-C-coated latex beads (red) incubated with RF/6A cells for 1 h with α-DNase X without permeabilization. Note a cluster of beads colocalizes with host cell-surface DNase X. Scale bar, 5 µm. (E) Selected time-lapse images (0 to 6:38 min) of rEtpE-C-coated beads attached to RF/6A cells expressing DNase X-GFP at 4°C, and time 0 min was set upon raising the temperature to 37°C. The white dashed line denotes the RF/6A cell contour. A single *z-*plane (0.4 µm thickness) by deconvolution microscopy was shown. Scale bar, 2 µm (see also Movie S1). (F) Line intensity profile analysis of red (rEtpE-C-beads) and green (DNase X-GFP) signal along the length of the line (slanted white line in the image 5E).

To confirm EtpE-C binding to the native human DNase X, we performed far-Western blot analysis. DNase X from the THP-1 cell lysate bound to re-natured rEtpE-C on a nitrocellulose membrane, whereas DNase X did not bind the control rECH0825 (Fig. 5A). Next, we utilized a protein pull-down assay wherein THP-1 cell lysate was applied to rEtpE-C bound to and renatured on a Ni-affinity matrix. Western blotting showed that native DNase X from the lysate bound to rEtpE-C, but not to the control rECH0825 (Fig. 5B). In addition, co-immunoprecipitation showed that anti-EtpE-C, but not the control mouse IgG pulled down native DNase X from the lysate of THP-1 cells incubated with *E. chaffeensis* for 30 min (Fig. 5C). Taken together, these results indicate that EtpE-C can bind to DNase X.

We fixed RF/6A or HEK293 cells incubated with rEtpE-C-coated beads, and without membrane permeabilization, immunofluorescence labeled cell surface-exposed DNase X. DNase X localized to the areas on the surface of cells where rEtpE-C-coated beads were present (RF/6A cell image shown in Fig. 5D). Time-lapse live-cell image analysis of rEtpE-C-coated beads bound to RF/6A cells ectopically expressing DNase X-GFP at 4°C showed that, upon warming up to 37°C, the initially separated DNase X-GFP signal and beads became closer and overlapped within 5 min (Fig. 5E; see also suppl. Movie S1). The fluorescence intensity profile analysis of red (rEtpE-C-coated beads) and green (DNase X-GFP) signal along the length of the line also revealed that the signals separated at initial time points converged in a few min after warming-up (Fig. 5F).

### Binding and internalization of rEtpE-C-coated beads is dependent on DNase X

**Figure 6:**
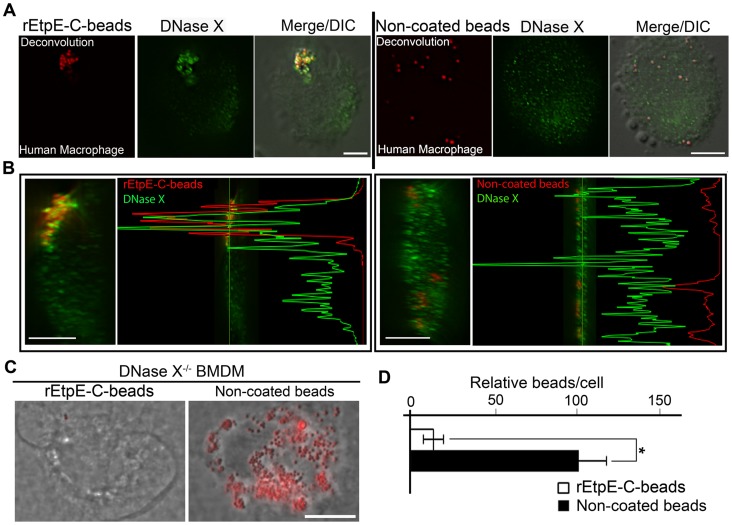
Internalization of rEtpE-C-coated beads is dependent on DNase X. (A) Immunofluorescence labeling of rEtpE-C-coated or non-coated beads incubated with human macrophages derived from peripheral blood monocytes. At 30 min pi, cells were labeled with α-DNase X without permeabilization. rEtpE-C-coated beads cluster and colocalize with DNase X on the cell surface, but non-coated beads do not. A single *z-*plane (0.4 µm thickness) by deconvolution microscopy was shown. Scale bar, 5 µm (see also suppl. Fig. S7 and suppl. Movie S2 and S3). (B) A selected image showing the orthogonal view of macrophage incubated with rEtpE-C-coated (left panel) or non-coated (right panel) beads in (A). The orthogonal view was obtained from the reconstituted 3-D view of serial *z*-stack images (combined z-section width of 7.2 µm). Scale bar, 5 µm. The fluorescence intensity profiles of green (DNase X) and red (beads) signals were shown. (C) Fluorescence and phase contrast merged images of rEtpE-C-coated and non-coated beads incubated with BMDMs from DNase X^−/−^ and wild-type mice. Cells and beads were incubated for 45 min followed by trypsin treatment to remove non-internalized beads. Scale bar, 10 µm. (D) Numbers of internalized rEtpE-C-coated beads/cell of similar experiment as (C), relative to the number of non-coated beads set as 100. Data represent the mean and standard deviation of triplicate samples and are representative of three independent experiments. *Significantly different (*P*<0.05) (see also suppl. Fig. S8).

Since DNase X localized to EtpE-C-coated beads in non-phagocytes, we next examined this phenomenon in phagocytes. Human primary macrophages derived from peripheral blood monocytes were incubated with rEtpE-C-coated or non-coated beads, cell surface exposed DNase X was immunofluorescence-labeled without permeabilization and the distribution of beads and DNase X was examined by deconvolution microscopy. Surface DNase X was seen clustered with rEtpE-C-coated beads; whereas both surface DNase X and beads were randomly dispersed with non-coated beads (image in a single *z-*plane shown in Fig. 6A). Orthogonal views of the cell from the reconstructed 3D view of serial z-stack images unequivocally demonstrated colocalization of DNase X with rEtpE-C-coated beads (Fig. 6B left panel, see also 3D view in suppl. Movie S2), whereas DNase X did not colocalize with non-coated beads (Fig. 6B right panel, see also 3D view in suppl. Movie S3). The intensity profile analysis of green (DNase X) and red (beads) signals of a single optical section showed that DNase X coincided with rEtpE-C-coated beads, but not with non-coated beads (Fig. 6B right panels). Similar results were observed with canine primary macrophages derived from peripheral blood monocytes and DH82 cells (suppl. Fig. S8). These results indicate DNase X localizes to rEtpE-C-coated beads in primary human and canine macrophages, the pathogen's *in vivo* target cells.

rEtpE-C-coated beads entered wild-type mouse BMDMs as shown in Figs. 3B and 3C. Therefore, we next examined whether rEtpE-C-coated beads can enter BMDMs from congenic DNase X^−/−^ mice. Beads were incubated with BMDMs from DNase X^−/−^ mice for 45 min followed by trypsin treatment to remove beads that were not internalized. Results showed rEtpE-C-coated beads did not enter DNase X^−/−^ BMDMs (Figs. 6C and 6D). In striking contrast, non-coated beads freely entered DNase X^−/−^ BMDMs (Figs. 6C and 6D). This lack of entry of rEtpE-C-coated beads into DNase X^−/−^ BMDM, but not into the wild-type BMDM, was a direct consequence of its failure to bind DNase X^−/−^ BMDM (suppl. Fig. S9). This phenomenon was specific to rEtpE-C coated beads, because neither the non-coated beads nor the rECH0825-coated beads showed any defect in binding DNase X^−/−^ BMDMs (suppl. Fig. S9). Taken together, these results indicate that rEtpE-C coating dictates the latex bead binding and entry via DNase X-dependent pathway.

### DNase X mediates *E. chaffeensis* binding, entry, and infection

**Figure 7:**
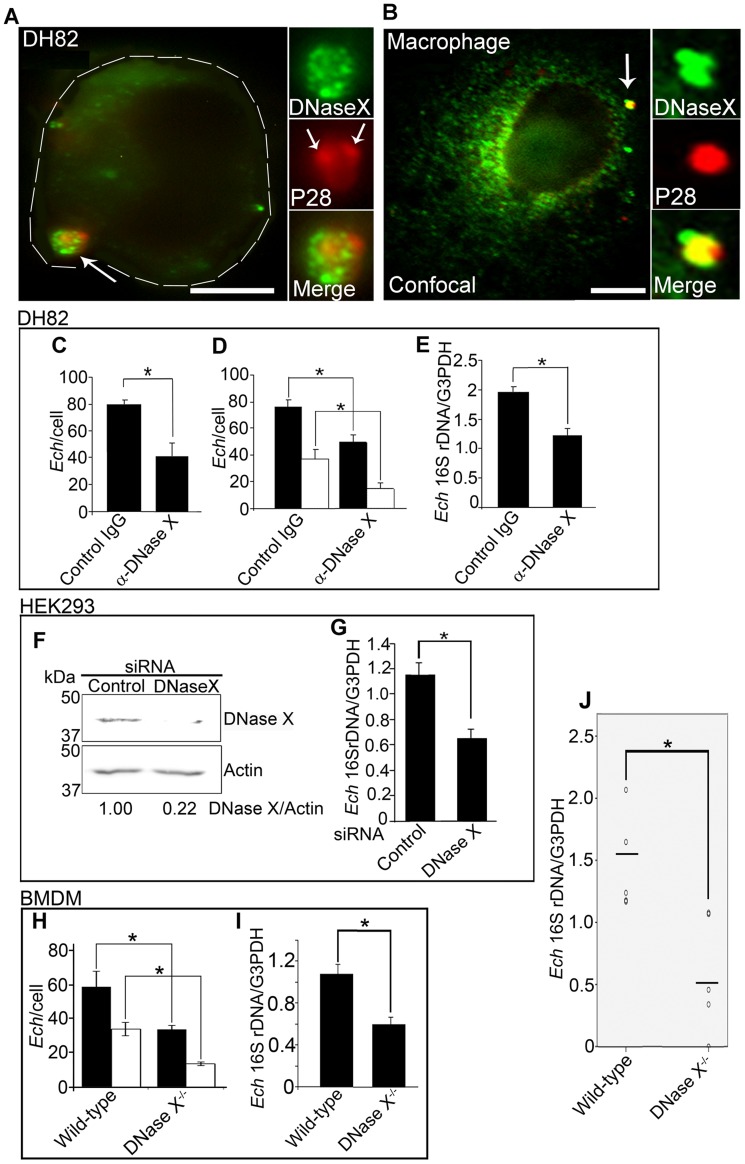
DNase X mediates *E. chaffeensis* binding, entry, and infection. (A) Double immunofluorescence labeling with α-P28 and α-DNase X, without permeabilization, of *E. chaffeensis* bound on DH82 cells at 45 min pi at MOI of 10∶1. The white dashed line denotes the DH82 cell contour. The arrow indicates the area enlarged in the smaller panels to the right. DNase X at the host-cell surface clusters to bound *E. chaffeensis* (arrows). Scale bar, 5 µm. (B) Confocal image of double immunofluorescence labeled *E. chaffeensis* on human macrophages derived from peripheral blood monocytes at 30 min pi at MOI of 10∶1, with α-P28 and α-DNase X without permeabilization. DNase X colocalizes with the sites of *E. chaffeensis* binding (arrow, the region enlarged in the smaller panels to the right). Scale bar, 5 µm. (C) Numbers of *E chaffeensis* bound to DH82 cells pretreated with α-DNase X or mouse IgG at 30 min pi. Immunofluorescence labeling with α-P28 was performed without permeabilization and the numbers of *E. chaffeensis* on 100 cells were scored. Data represent the mean and standard deviation of triplicate samples and are representative of three independent experiments. *Significantly different (*P*<0.05). (D) Numbers of *E. chaffeensis* internalized into DH82 cells pretreated with α-DNase X or mouse IgG at 2 h pi. Cells were processed for two rounds of immunostaining with α-P28 as described in Fig. 1E. The black bar represents total *E. chaffeensis*, and the white bar represents internalized *E. chaffeensis* (total minus bound). *E. chaffeensis* in 100 cells were scored. Data represent the mean and standard deviation of triplicate samples and are representative of three independent experiments. *Significantly different (*P*<0.05). (E) *E. chaffeensis* load in DH82 cells pretreated with α-DNase X or mouse IgG at 48 h pi. qPCR for *E. chaffeensis* 16S rDNA normalized with canine G3PDH DNA. Data represent the mean and standard deviation of triplicate samples and are representative of three independent experiments. *Significantly different (*P*<0.05). (F) Western blot analysis of DNase X in HEK293 cells transfected with DNase X siRNA or scrambled control siRNA. Actin was used as a protein loading control. (G) *E. chaffeensis* load in HEK293 cells treated with DNase X siRNA or scrambled control siRNA at 48 h pi. qPCR for *E. chaffeensis* 16S rDNA normalized with human G3PDH DNA. Data represent the mean and standard deviation of triplicate samples and are representative of three independent experiments. *Significantly different (*P*<0.05). (H) Bar graph showing numbers of total cell-associated and internalized *E. chaffeensis* in DNase X^−/−^ or wild-type BMDMs at 4 h pi. Cells were processed for two rounds of immunostaining with α-P28 as described in Fig. 1E. The total numbers of *E. chaffeensis* in 100 cells were scored. Data represent the mean and standard deviation of triplicate samples and are representative of three independent experiments. The black bar represents total *E. chaffeensis*, and the white bar represents internalized *E. chaffeensis* (total minus external). *Significantly different (*P*<0.05) (I and J) *E. chaffeensis* load in BMDMs from DNase X^−/−^ mice and wild-type mice at 56 h pi (I) or in the blood at 5 days post-infection from DNase X^−/−^ mice and wild-type mice (J). qPCR for *E. chaffeensis* 16S rDNA was performed and normalized with mouse G3PDH DNA. Data represent the mean and standard deviation of triplicate samples and are representative of three independent experiments. *Significantly different (*P*<0.05).

Since DNase X was localized to EtpE-C-coated bead entry foci, we next examined whether DNase X localizes to the *E. chaffeensis* entry foci as well. Double immunofluorescence labeling of non-permeabilized DH82 cells and primary human macrophages derived from human peripheral blood monocytes showed surface DNase X colocalization with the bound *E. chaffeensis* (Figs. 7A and 7B). DNase X also localized to the areas of *E. chaffeensis* binding on THP-1 cells (data not shown). When DH82 cells were pre-incubated with monoclonal anti-DNase X IgG to block the surface-exposed DNase X, *E. chaffeensis* binding, entry, and overall infection were significantly reduced compared with the control mouse IgG-treated DH82 cells (Figs. 7C–7E). Next, we utilized a small interfering RNA (siRNA) against DNase X to reduce the expression of endogenous DNase X in HEK293 cells (Fig. 7F). Suppression of DNase X expression significantly reduced *E. chaffeensis* infection in HEK293 cells (Fig. 7G). Moreover, *E. chaffeensis* binding and entry were reduced by 60% in DNase X^−/−^ BMDMs compared to the wild-type BMDMs (Fig. 7H). *E. chaffeensis* load at 56 h pi was significantly lower in DNase X^−/−^ BMDMs compared to the wild-type BMDMs (Fig. 7I). These results demonstrated that effective *E. chaffeensis* binding, entry, and infection depended on DNase X. Importantly, *E. chaffeensis* load in peripheral blood at 5 days pi in DNase X^−/−^ mice was significantly lower than in wild-type mice (Fig. 7J), indicating that effective *in vivo* infection of *E. chaffeensis* also requires involvement of DNase X.

## Discussion

*E. chaffeensis* entry of permissive host cells is an absolute requisite, not only in the pathogenesis of HME, but also for the very existence of the bacterium in nature owing to its inability to survive outside of eukaryotic cells. The present study showed that *E. chaffeensis* surface protein EtpE is not only an adhesin that binds a specific receptor DNase X, a GPI-anchored mammalian cell surface protein, but also an invasin that subsequently mediates bacterial internalization. *E. chaffeensis* entry into phagocytes and non-phagocytes was similar, but completely different from the phagocytosis of non-coated latex beads. Differences include 1) ability of *E. chaffeensis* to bind and enter non-phagocytes vs. inability of non-coated beads to enter non-phagocytes, 2) inhibition of *E. chaffeensis* entry into phagocytes and non-phagocytes by MDC, genistein, verapamil, and PI-PLC vs. lack of inhibition of entry of non-coated beads into phagocytes by these compounds, 3) DNase X-dependent entry of *E. chaffeensis* into phagocytes and non-phagocytes, vs. DNase X-independent entry of non-coated beads into phagocytes, and 4) colocalization of DNase X and *E. chaffeensis* during entry vs. lack of colocalization of non-coated beads with DNase X. Remarkably, coating with a fragment of a single protein, EtpE-C made the beads to bind and enter non-phagocytes and phagocytes like *E. chaffeensis* does. Similarities between EtpE-C-coated beads and *E. chaffeensis* include 1) signaling pathways for invasion sensitive to MDC, genistein, verapamil, and PI-PLC, 2) DNase X-dependent adhesion and invasion, 3) colocalization with DNase X at entry foci during invasion, and 4) ultrastructure of entry process (filopodia, [4]). Some of these features are summarized in Table 1.

**Table 1:** Summary- Internalization of *E. chaffeensis*, rEtpE-C-coated or non-coated beads.

Candidate	Non-phagocytes^1^	Phagocytes^2^	DNase X^−/−^BMDMs	Drug^3^-pretreated host cells
***E. chaffeensis***	Internalized	Internalized	Poorly Internalized	Not Internalized
**rEtpE-C-coated beads**	Internalized	Internalized	Not Internalized	Not Internalized
**Non-coated beads**	Not Internalized	Internalized	Internalized	Internalized

1 HEK293 and RF/6A cells.

2 Human primary macrophages derived from blood monocytes, Canine primary macrophages derived from blood monocytes, DH82 cells, THP-1 cells, and mouse bone-marrow-derived macrophages (BMDMs).

3 MDC, genistein, verapamil, or PI-PLC.

Our results demonstrate that in the absence of any other bacterial proteins EtpE-C is sufficient to orchestrate the binding and entry of coated beads, and in extension *E. chaffeensis* binding and entry, into its host cells. Protein coating of latex beads has been widely used for phagocytosis study [34] and also been previously utilized for studies with *Listeria* internalin [35]. For studying rickettsial adhesins and invasins, *Escherichia coli-*based heterologous protein expression system has been utilized [36], [37]. Using latex beads coated with rickettsial proteins provides an alternative way to study rickettsial invasin. The method is especially useful for those proteins poorly expressed on *E. coli* surface and/or when using host cells, such as macrophages, that are easily perturbed by *E. coli*.

Function of EtpE-C as an *E. chaffeensis* invasin mediating its DNase X-dependent entry of host cells is strongly supported by *in vitro* and *in vivo* anti-EtpE-C neutralization results, and *in vitro* and *in vivo* requirement of DNase X for efficient *E. chaffeensis* infection. The results suggest *E. chaffeensis* invasion process, like inert rEtpE-C-coated latex beads, does not require bacterial energy. Adhesion alone seems to be sufficient in triggering cellular receptor-mediated signaling pathways resulting in filopodial extension and internalization. This is distinct from the triggering of bacterial uptake by the action of actively injected bacterial type III secretion system effector molecules [38], [39], but in agreement with the condensed and resistant features of *E. chaffeensis* DCs capable of invasion [18]. This also is in agreement with the regulation of EtpE expression by a DNA binding protein BolA, which is in turn regulated by CtrA, a response regulator of the two-component system [18]. *E. chaffeensis* CtrA positively regulates genes involved in the development of the resistant phenotype, at the late stage of *E. chaffeensis* growth cycle, when RCs convert to DCs [18]. The rosary-like pattern of EtpE in host cell surface-bound *E. chaffeensis* did not preexist in host cell-free *E. chaffeensis*; thus might be induced by its engagement with cell surface DNase X upon binding. Significance of this change in EtpE surface distribution awaits further investigation.

Although both EtpE-N and EtpE-C were conserved among *E. chaffeensis* strains, only EtpE-C mediated binding and entry into non-phagocytes, indicating functional specificity of this segment. In addition to the poor accessibility of anti-EtpE-N to its target on live *E. chaffeensis* surface, the inability of EtpE-N to function as an invasin seems to be the primary reason for the weak *in vitro* neutralization of *E. chaffeensis* infection with anti-EtpE-N. While both EtpE-N and EtpE-C fragments were recognized and elicited humoral immune response in naturally infected humans and experimentally infected dogs, it seems that the antibody titer to EtpE-C is lower than to EtpE-N. This lower titer may help *E. chaffeensis* to establish infection. Although the present study did not probe the function of the central region of EtpE, being variable among *E. chaffeensis* strains, this segment of the protein may be under selective pressure in the host for *E. chaffeensis* to persist in nature. Although EtpE is found in all *Ehrlichia* spp., amino acid sequences corresponding to EtpE-C are not conserved among EtpE orthologs of *Ehrlichia* spp.; whether these orthologs also serve as adhesins/invasins in other *Ehrlichia* sp. remain to be studied.

The present study showed that not only antibody against rEtpE-C neutralizes *E. chaffeensis* infection *in vitro*, but also immunization of mice with rEtpE-C confers protection against *E. chaffeensis* challenge. It has been reported that adoptive transfer of immune serum from immunocompetent C57BL/6 mice to immuno-compromised SCID mice confers significant protection from fatal *E. chaffeensis* infection [36]. Mice with depleted complements, or lacking B cells, FcγR1, CR1, CR2, or Nox2 (a subunit of NADPH oxidase) are more susceptible to non-lethal dose infection with the HF strain of *Ehrlichia* (*Ixodes ovatus Ehrlichia*) that is closely related to *E. chaffeensis* [28]. *N. risticii* infects P388D_1_ macrophages, but FcR-mediated entry kills this bacterium and Fab fragment of the immune IgG blocks binding and entry of *N. risticii* [37]. Whether any of these mechanisms are involved in protection of mice from *E. chaffeensis* infection by rEtpE-C immunization awaits further study. Nonetheless, our results from *in vitro* neutralization studies suggest a direct inhibition of *E. chaffeensis* binding by anti-EtpE-C antibody and support the importance of antibodies in ehrlichial immunity. A recent study showed that EtpE mRNA is highly expressed by *E. chaffeensis* in cell lines derived from the ticks, *Amblyomma americanum* and *Ixodes scapularis* using *E. chaffeensis* genome microarrays [38]. Since *E. chaffeensis* is transmitted between mammals by tick bite, expression of EtpE in tick cells suggests an indispensable role for this protein throughout the *E. chaffeensis* life cycle.

DNase X-mediated uptake is the first entry pathway to be uncovered for *Ehrlichia* spp. Compared to DNase I, DNase X has an extra hydrophobic stretch at its C-terminus [32]. This C-terminal stretch is conserved in all mammalian DNase X proteins examined, and required for GPI-anchoring to the plasma membrane [33], [40]. Cellular functions of DNase X are not understood well. There is no obvious defect in DNase X^−/−^ mice and their reproduction is normal [41]. DNase X was never reported to serve as a receptor for any infectious agents. –Overexpression of DNase X enhances degradation of the exogenous plasmid DNA, consequently suppressing transformation in RD myotubes; whereas siRNA-mediated DNase X knockdown reverses this inhibition [33]. DNase X is, therefore, considered as a barrier in naked DNA transfer [33].

rEtpE-C-coated beads, unlike non-coated beads or rECH0825-coated beads, were unable to bind or enter DNase X^−/−^ BMDMs. Un-opsonized non-coated latex beads are taken up by macrophages predominantly via scavenger receptors [42]–[44]. This suggests that rEtpE-C-coating not only made beads to bind DNase X, but also to repel or avoid the scavenger receptor recognition. The detailed mechanism underlying this phenomenon remains to be clarified.

**Figure 8:**
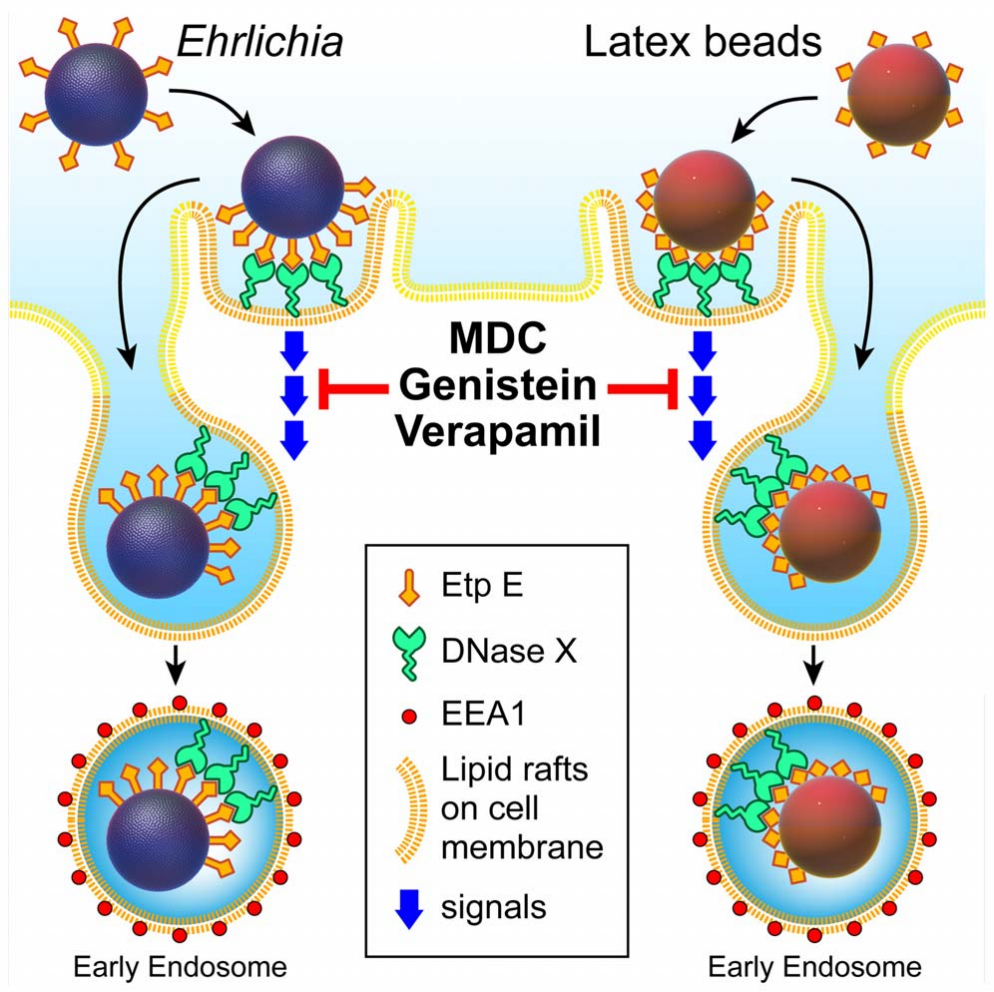
Schematic representation of *E. chaffeensis* binding and entry into mammalian cells. DNase X is enriched in the lipid raft domains of the cell membrane. Extracellular *E. chaffeensis* uses its surface protein EtpE C-terminal region to make initial contacts with cell surface DNase X that results in further lateral redistribution and local clustering of DNaseX at the sites of bacterial binding. This binding elicits signals that are relayed down-stream and culminated in host cytoskeletal remodeling, filopodial induction and engulfment of the bound bacteria into an early endosome into the host cell. This receptor-mediated endocytosis can be specifically disrupted by genistein, verapamil or MDC. Latex beads coated with rEtpE-C also bind to cell surface DNase X and follows a similar pattern of entry like that of *E. chaffeensis*.

The EtpE-DNase X-mediated entry of host cells by *E. chaffeensis* seems to be by a ‘zipper mechanism’ [39] initiated by specific contacts between bacterial ligand and host cell surface receptor and sequential engagement of the host cell surface membrane against bacterial surface. A schematic representation of a working model for *E. chaffeensis* binding and invasion of its target cells is depicted in Fig. 8. The initial binding and engagement of DNase X in the lipid-raft enriched areas on the host cell surface by EtpE C-terminal region causes clustering and lateral re-distribution of DNase X molecules to the site of *E. chaffeensis* attachment. This binding elicits signals that culminate in host cytoskeletal remodeling, filopodial formation embracing the bacteria and engulfment of the bound bacteria into an early endosome in the host cell. This receptor-mediated endocytosis can be specifically disrupted by genistein, verapamil or MDC.

Among members of the order Rickettsiales, the bacterial ligand and the cognitive host cell receptor pair for invasion have been reported only for *Rickettsia conorii* using *in vitro* cultured cells. For invasion, *R. conorii* uses an autotransporter protein OmpB as the ligand and Ku70, one of the DNA-binding components of the DNA-dependent protein kinase of mammalian cells as the receptor [45]. Whether Ku70 is required for *in vivo* infection by *Rickettsia* spp. is unknown. OmpB was originally identified as an invasive rickettsial protein capable of mediating binding and entry of host cells in *R. japonica*, another spotted fever group *Rickettsia* closely related to *R. conorii* [36]. *Anaplasma phagocytophilum*, the causative agent of human granulocytic anaplasmosis, binds to fucosylated P-selectin glycoprotein ligand-1 (PSGL-1) as a key initial receptor component [46]. α1,3-fucose is critical for *A. phagocytophilum* to infect neutrophils in mice and for the bacterium to colonize ticks [47], [48]. α2,3-sialic acid at the PSGL-1 N-terminus was identified as the receptor for an *A. phagocytophilum* adhesin, the peptidoglycan-associated lipoprotein, OmpA [49]. The present study identifies the first *Ehrlichia* invasin-receptor pair and is a critical advancement as this provides the first evidence for the importance of a given invasin-receptor pair *in vivo* for any pathogens belonging to the order Rickettsiales. In contrast to the almost complete abrogation of entry of rEtpE-C-coated beads into DNase X^−/−^ BMDMs, *E. chaffeensis* entry into DNase X^−/−^ BMDMs was reduced by 60% compared to wild-type BMDMs. This suggests existence of additional mammalian receptors for *E. chaffeensis* infection. Similarly *R. conorii* invasion of embryonic fibroblasts derived from Ku70^−/−^ mice is reduced by 50% compared to the cells derived from the wild-type litter mates [45]. A recent report has shown that *E. coli* expressing another autotransporter protein of *R. conorii*, OmpA, can invade endothelial cells by interacting with α2β1 integrin [50]. Rickettsial OmpA was originally suggested as an adhesin in *R. rickettsii* [51]. Although *Rickettsia* and *Ehrlichia* spp. are phylogenetically related (both belong to the order Rickettsiales), and share similar wild animal reservoirs and vector ticks in nature, *E. chaffeensis* EtpE and *R. conorii* invasin OmpB/OmpA are uniquely evolved in them, respectively [52]. This perhaps enabled these bacteria to utilize independent host surface receptors: DNase X and Ku70, respectively, for invasion to infect broader host range and cytoplasmic niche.

In light of severe and potentially fatal outcomes of HME, the limited choice of antibiotics and lack of prophylactic measures, further understanding of invasion mechanisms is of great importance. Such information will assist in the development of new preventive and therapeutic measures against HME and similar diseases by specific pharmacological or immunologic disruption of invasin and the host cell receptor interaction.

## Materials and Methods

### Ethics statement

All animal experiments were performed in accordance with The Ohio State University Institutional Animal Care and Use Committee guidelines and approved e-protocol numbers 2009A0186 and 2008A0066. The University program has Full Continued Accreditation by the Association for Assessment and Accreditation of Laboratory Animal Care International (AAALAC-I) under #000028 dated June 9, 2000 and has a Public Health Services assurance renewal #A3261-01 dated February 5, 2007 through February 28, 2015. The program is licensed by the USDA, #1-R-014, and is in full compliance with Animal Welfare Regulations.

### *E. chaffeensis* and host cell culture

*E. chaffeensis* Arkansas type strain was propagated in DH82 cells, and host cell–free *E. chaffeensis* was obtained by controlled sonication as described [53], [54]. Human peripheral blood monocytes were derived from buffy coats; HEK293, RF/6A and THP-1 cells were cultured as previously described [7], [8], [54]. BMDMs were established from wild-type and DNase X ^−/−^ mice as described [8].

### Mouse infection studies

Two groups of C3H/HeJ mice (4-week-old females; 4 mice per group) (Jackson Laboratories) received either minced SDS-polyacrylamide gel containing 50 µg of rEtpE-C or minced gel alone, with Quil A (Accurate Chemicals) as adjuvant for a total of three times at 14-day intervals. *E. chaffeensis* challenge was performed 10 days following the last immunization as described [21]. DNase X^−/−^ [41] and congenic wild-type C57BL/6 mice (5- to 6-week-old females; 5 mice per group) were inoculated intraperitoneally with *E. chaffeensis*-infected THP-1 cells (>90% cells infected; 6×10^5^ cells/mouse). DNA was extracted from blood samples using a QIAamp blood kit (Qiagen), and subjected to qPCR using *E. chaffeensis* 16S rDNA and mouse glyceraldehyde 3-phosphate dehydrogenase (G3PDH) gene primers.

### Production of recombinant proteins rEtpE-N and rEtpE-C, and antisera against them

DNA fragments encoding EtpE-C and EtpE-N were amplified by PCR with Phusion high-fidelity DNA polymerase (NEB) using *E. chaffeensis* chromosomal DNA as template. The fragments were cloned into pET33b(+) vector (Novagen); recombinant proteins were expressed in *E. coli* BL21 (DE3) and purified by Ni-affinity chromatography. The antibody against rEtpE-C was produced in ICR mice (Harlan), and the antibody against rEtpE-N was produced in rabbits.

### Western blot analysis with sera from HME patients and *E. chaffeensis*-infected dogs

Affinity-purified rEtpE-C and rEtpE-N (5 µg each) were subjected to SDS-PAGE, transferred to a nitrocellulose membrane, and incubated with sera from *E. chaffeensis*-infected dogs (CTUALJ, 3918815, 1425) [28], HME patients (ID: MRL1-22, MRL1-40, 72088) [27], or control sera. After washing, the membranes were incubated with horseradish peroxidase–conjugated goat anti-dog or anti-human IgG (KPL). Reacting bands were visualized with enhanced chemiluminescence (ECL), images were captured and densitometric analysis was performed using an LAS3000 image documentation system (FUJIFILM Medical Systems).

### Yeast two-hybrid screening

Yeast two-hybrid screening was performed using Matchmaker Two-Hybrid System (Clontech) according to manufacturer's instructions. The bait plasmid pGBKT7-EtpE-C was constructed by the fusion of EtpE-C with the GAL4 DNA-binding domain in pGBKT7 (Clontech) by PCR. EtpE-C coding sequence was amplified using the forward primer 5′-AATCCATGGAATTGTTGTCATTAGTTGGTGGGCATCG-3′ (5′ NcoI site underlined) and reverse primer 5′-TCGACGGATCCAATCCCCTTCCAGCATTAATTTTATCAAAGG-3′ (5′ BamHI site underlined), and the product was ligated into pGBKT7. pGBKT7-EtpE-C was transformed into *Saccharomyces cerevisiae* strain AH109 and selected by the ability to grow on SD agar plates lacking tryptophan. The expression of bait protein EtpE-C in yeast was examined by Western blotting. The human bone marrow MATCHMAKER cDNA library (Clontech) that was fused with GAL4-activating domain in pGADT7 was transformed in *S. cerevisiae* strain Y187 (Clontech). Library clones expressing interacting prey proteins were screened with yeast mating. Positive clones were selected by their ability to grow on SD quadruple drop-out (SD/QDO) plates lacking adenine, histidine, leucine, and tryptophan, and verified on SD/QDO plates containing X-gal. Positive clones were then isolated, and the prey plasmids were purified and sequenced after they were transformed into *E. coli* TOP10F′ competent cells (Invitrogen). The interaction was confirmed by re-shuttling the purified prey plasmid into *S. cerevisiae* AH109 transformed with bait plasmid and by nutritional selection in SD/QDO plates.

### Binding and internalization assay of *E. chaffeensis* and immunostaining of host cell-free *E. chaffeensis*

Coverslip cultures of DH82, HEK293, RF/6A cells, or macrophages differentiated from human peripheral blood monocytes or established from bone-marrow of DNase X^−/−^ or congenic wild-type mice and suspension culture of THP-1 cells were incubated with *E. chaffeensis* freshly isolated from infected cells at approximate multiplicity of infection (MOI) of 200, unless otherwise noted, for 30 to 45 min for binding assays or 2 to 4 h for internalization assays at 37°C in 5% CO_2_/95% air. Cells were washed with phosphate-buffered saline (PBS: 137 mM NaCl, 2.7 mM KCl, 8.1 mM Na_2_HPO_4_, 1 mM KH_2_PO_4_, pH 7.4) to remove unbound bacteria and labeled with antibodies as described [23]. For binding assay cells were fixed with 3% PFA and labeled with mouse monoclonal anti-DNase X (Abcam), rabbit anti-*E. chaffeensis* P28 [21], mouse anti-rEtpE-C, or dog anti-*E. chaffeensis* [55] as primary antibodies and Alexa Fluor (AF) 488–conjugated goat anti-mouse IgG, AF555–conjugated goat anti-rabbit IgG (Invitrogen), or Texas Red–conjugated goat anti-dog IgG (Jackson ImmunoLab) as secondary antibodies. For internalization assays, two steps of labeling of fixed cells with anti-P28 were carried out as described: the first labeling step was performed without saponin permeabilization using AF488–conjugated goat anti-rabbit IgG, and the second labeling was performed with permeabilization using AF555–conjugated goat anti-rabbit IgG [56]. Fluorescent images were acquired using a Nikon Eclipse E400 fluorescence microscope with a xenon-mercury light source (Nikon), Deltavision deconvolution microscope (Applied Precision) with 0.2 or 0.4-µm step size along the *z*-axis of the cells, or an LSM 510 laser-scanning confocal microscope (Carl Zeiss). For immunostaining of live bacteria, host cell-free *E. chaffeensis* was incubated with anti-EtpE-C, anti-EtpE-N or *E. chaffeensis* P28 for 1 h at room temperature followed by fixing with 3% PFA and labeling with AF555-conjugated goat anti-mouse or anti-rabbit antibodies. To further demonstrate the surface exposure of EtpE, host cell-free *E. chaffeensis* was incubated with either pronase E (Sigma) at a concentration of 2 mg/ml in PBS or vehicle control for 15 min at 37°C [57]; pronase E was inactivated by adding 10% fetal bovine serum (FBS), followed by washing in PBS twice. The bacteria were cytospun onto glass slides, fixed with 3% PFA, followed by quenching in PBS containing 0.1 M glycine, washed with PBS and labeled sequentially with anti-EtpE-C and anti-CtrA [18] with or without saponin permeabilization followed by AF488 or AF555-conjugated goat anti-mouse or anti-rabbit antibodies.

### [^35^S]methionine-labeled *E. chaffeensis* binding and uptake

Approximately 10^6^ cells of *E. chaffeensis*-infected THP-1 cells/ml in 2 ml of methionine cysteine-deficient RPMI 1640 medium (ICN Biomedicals) supplemented with 10% FBS and 2 mM l-Gln were incubated with cycloheximide (Sigma) at 10 µg/ml at 37°C for 1 h. A metabolic labeling reagent (Tran ^35^S-Label; 11.7 mCi/ml [1,100 Ci/mmol]; 100 µl; ICN Biomedicals) was added and the mixture was incubated further at 37°C for 24 h. The radiolabeled *E. chaffeensis* was released by sonication and washed by centrifugation at 10,000×g for 10 min. To study the effect of rabbit anti-P28 on *E. chaffeensis* binding and entry, radiolabeled *E. chaffeensis* cells (40,000 cpm/200 µl) preincubated with Fab fragment of anti-P28 IgG [0.5 mg/ml, prepared using Immobilized papain (Pierce) from IgG affinity purified with AffiPack Immobilized Protein A column (Pierce)] or Fab fragment of normal rabbit IgG (0.5 mg/ml) were added to 1×10^6^ THP-1 cells in 0.4 ml of RPMI 1640 medium containing 10% FBS and 2 mM l-Gln and incubated at 4°C for 2 h. The uptake of *E. chaffeensis* was evaluated following removal of bound *E. chaffeensis* cells by incubation with pronase E at 2 mg/ml in PBS at 37°C for 10 min after incubation of *E. chaffeensis* with THP-1 cells at 37°C for 3 h. THP-1 cells were washed by centrifugation at 375×g for 5 min, the cells then were dissolved in 0.6 N NaOH and 0.5% SDS, and the radioactivity was measured in a scintillation counter.

### *In vitro* neutralization and RNA interference

*E. chaffeensis* preincubated with 25 µg/ml of mouse anti-rEtpE-C, rabbit anti-rEtpE-N, or preimmune mouse or rabbit sera for 1 h at 4°C were used to infect THP-1, RF/6A, or DH82 cells. Alternatively, *E. chaffeensis* was added to DH82 cells preincubated with 10 µg/ml of monoclonal anti-DNase X or control mouse monoclonal antibody for 30 min at 25°C in serum-free DMEM. Binding, internalization, and infection were determined at 30 min, 2 h and 48 h pi, respectively. HEK293 cells in 24-well plates were transfected with 50 nM DNase X siRNA (Santa Cruz Biotechnology) or scrambled control siRNA using Lipofectamine 2000 (Invitrogen). A second transfection with 50 nM of DNase X and scrambled siRNAs was performed 30 h after the first transfection. An aliquot of cells were harvested at 24 h after the second transfection to determine the protein amount of DNase X by Western blotting and densitometry analysis with anti-DNase X and rabbit anti-actin (Sigma). The other aliquot of cells were incubated with *E. chaffeensis* and incubated for an additional 48 h to evaluate infection. Infection was determined by qPCR of *E. chaffeensis* 16S rRNA gene relative to host cell G3PDH gene [18].

### Far-Western blotting, protein affinity pull-down and co-immunoprecipitation

Far-Western blotting was performed using 5 µg of rEtpE-C and rECH0825 that were separated by SDS-PAGE, transferred to a nitrocellulose membrane and renatured with serial guanidinium-HCl treatment followed by incubation with THP-1 cell lysate in NP-40 lysis buffer (150 mM NaCl, 50 mM Tris-HCl pH 7.4, 1% w/v NP-40, supplemented with 1% protease inhibitor cocktail set III [Calbiochem]) as described [58]. After stringent washing, the membrane was incubated with anti-DNase X and peroxidase-conjugated goat anti-mouse antibodies (KPL). The membrane was stripped with Restore Western Blot Stripping Buffer (Thermo scientific) and reprobed with peroxidase-conjugated anti-histidine antibody (Sigma). For protein pull-down, His-tagged rEtpE-C was bound to and renatured on the Ni-affinity matrix (Promega). THP-1 cell lysate in NP-40 lysis buffer was applied to the matrix and incubated for 8 h at 4°C. After washing off the unbound or non-specifically bound proteins from the matrix, rEtpE-C and bound protein complex were eluted with 250 mM imidazole. The eluate and the post-elution Ni-matrix were resuspended in 2× SDS-sample buffer and subjected to Western blotting with anti-DNase X antibody. For co-immunoprecipitation assay, THP-1 cells were incubated with *E. chaffeensis* for 30 min and lysed in NP-40 lysis buffer. The lysate was immunoprecipitated with anti-EtpE-C (2 µg)-bound protein A agarose or control mouse IgG (2 µg)-bound agarose beads. The precipitate was resuspended in 2× SDS-sample buffer and subjected to Western blotting with anti-DNase X antibody.

### Binding and internalization of latex beads

Sulfate-modified fluorescent red polystyrene beads (0.5 µm diameter; Sigma) at 3–4×10^6^ beads in 200 µl of 25 mM 2-(N-morpholino)ethanesulfonic acid (MES) buffer, pH 8.0 were incubated with 1 µg of rEtpE-C or rEtpE-N proteins in 5–7 µl 7 M urea in 50 mM sodium phosphate buffer, pH 7.2 at 4°C overnight with mixing at 20 rpm. MES buffer (150 µl) was sequentially added to the mixture every 15 min and incubated at room temperature, rotating at 20 rpm eventually diluting to around 200 times the original volume of urea buffer. rECH0825 and rGroEL were treated similarly, but without urea. The coated beads were collected by low speed centrifugation, washed twice in MES buffer and resuspended in complete DMEM or advanced MEM media, then gently sonicated to disperse the beads. Protein coating of the beads were confirmed by dot blot assay and/or immunofluorescence labeling. Freshly prepared protein-coated or non-coated beads were added at a multiplicity of approximately 50 beads per cell for co-localization studies and 500 beads per cell for quantitation of binding and internalization studies. The beads were incubated with HEK293 or RF/6A cells for 1 h at 37°C. Unbound beads were removed by washing and cells were fixed for immunofluorescence labeling to detect the localization of DNase X or EEA1 (anti-EEA1, BD). To study the effect of MDC, genistein, or verapamil on bead internalization, RF/6A cells were incubated with these chemicals at a final concentration of 100 µM or 0.1% DMSO solvent control for 30 min, then with rEtpE-C-coated beads for 8 h at 37°C. BMDMs from wild-type mice were pre-incubated with MDC or genistein (100 µM), PI-PLC (5 U/ml) or 0.1% DMSO for 30 min and then incubated with rEtpE-C-coated or non-coated beads for 45 min at 37°C. PI-PLC-treated cells were washed prior to addition of rEtpE-C-coated beads. The cells were washed and treated with 0.25% trypsin at 37°C for 10 min to remove surface-bound beads. The detached cells were further washed by low-speed centrifugation and later cytocentrifuged onto a glass slide and fixed with 3% PFA to observe internalized beads. To estimate the number of bound beads, a similar procedure for observing internalized beads was followed except that the beads were incubated with BMDM for 30 min at 4°C and following incubation the cells were washed to remove loosely bound beads and directly fixed with 3% PFA without trypsin treatment. For scanning electron microscopy, rEtpE-C-coated beads were incubated with RF/6A cells for 2 h at 37°C and processed as described previously [59]. For transmission electron microscopy, coated beads were incubated with RF/6A cells for 8 h at 37°C and processed as described previously [60]. The 3D orthogonal view of the cell to show spatial distribution of DNase X with beads was obtained by using the volume viewer function of SoftWoRx DeltaVision image acquisition software from Applied Precision.

### Live-cell imaging

RF/6A cells were cultured in 35-mm glass bottom dishes (Wilco), transfected with DNase X-GFP, and incubated with rEtpE-C-coated beads at 4°C for 1 h to facilitate bead binding, but prevent internalization. Unbound beads were washed off, cells were replenished with medium lacking phenol red, and the samples moved to a controlled environmental chamber at 37°C with under 5% CO_2_/95% air. Time-lapse images were acquired at an interval of 10 s for 5 to 20 min through a 60×1.42 NA oil immersion lens with an inverted Olympus IX-70 microscope, in 0.4-µm steps in the *z*-axis using the attached Applied Precision motorized stage (DeltaVision deconvolution microscope). All stacks of images were deconvoluted using SoftWoRx software and the time-lapse images of a single focal plane of 0.4-µm focal depth at the cell surface were exported as a video.

### Statistical analysis

Statistical analysis was performed by unpaired two-tailed Student's *t*-test. *P*<0.05 was considered to be significant.

## Supporting Information

Figure S1 **Alignment of Amino acid sequence of EtpE orthologs among three sequenced** ***Ehrlichia chaffeensis*** **strains, related to** **Fig. 1** **.** *E. chaffeensis* EtpEs of Arkansas, Wakulla, and Liberty (GenBank accession no. YP_507823.1, DQ915979.1 and DQ924562.1, respectively), were aligned by Clustal W using MegAlign. The red shade represents residues that match the consensus.
(TIF)

Figure S2 **Alignment of Amino acid sequence of EtpE orthologs among three sequenced** ***Ehrlichia*** **species, related to** **Fig. 1** **.** *E. chaffeensis* Arkansas EtpE, *E. canis* Jake Ecaj_0838 and *E. ruminantium* Welgevonden Erum7970 (GenBank accession no. YP_507823.1, AAZ68869.1 and YP_180660.1, respectively), were aligned by Clustal W using MegAlign. The red shade represents residues that match the consensus.
(TIF)

Figure S3 **EtpE is bacterial surface exposed, related to** **Fig. 1C** **.** Immunofluorescence image showing host cell-free *E. chaffeensis* that was either treated with pronase E or PBS control. Cells were processed for double immunostaining with anti-EtpE-C and anti-CtrA with or without saponin permeabilization as described to distinguish extracellular and internalized bacteria. When bacteria were treated with pronase E, the surface immunofluorescence staining of EtpE was abolished completely, but not that of the internal control CtrA. Scale bar, 1 µm.
(TIF)

Figure S4 **Anti-EtpE-C neutralizes** ***E. chaffeensis*** **binding and entry into THP-1 cells, related to** **Fig. 1D-F** **.** (A) Numbers of *E. chaffeensis* (*Ech*) bound to THP-1 cells at 30 min pi. *E. chaffeensis* was pretreated with anti-EtpE-C or preimmune mouse serum and incubated with THP-1 cells for 30 min. Unbound *E. chaffeensis* was washed away, cells were fixed with PFA and *E. chaffeensis* was labeled with anti-P28 without permeabilization. *E. chaffeensis* in 100 cells was scored. (B) Numbers of *E. chaffeensis* internalized into THP-1 cells at 2 h pi. Purified host cell-free *E. chaffeensis* was pretreated with anti-rEtpE-C or preimmune mouse serum and incubated with THP-1 cells for 2 h. To distinguish intracellular from bound *E. chaffeensis*, unbound *E. chaffeensis* was washed away, and cells were processed for two rounds of immunostaining with anti-P28: first without permeabilization to detect bound but not internalized *E. chaffeensis* (AF555–conjugated secondary antibody), and another round with saponin permeabilization to detect total *E. chaffeensis*, i.e., bound plus internalized (AF488–conjugated secondary antibody). The black bar represents total *E. chaffeensis*, and the white bar represents internalized *E. chaffeensis* (total minus bound). *E. chaffeensis* in 100 cells was scored. qPCR for *E. chaffeensis* 16S rDNA was normalized with human G3PDH DNA. Data represent the mean and standard deviation of triplicate samples and are representative of three independent experiments. *Significantly different (*P*<0.05).
(TIF)

Figure S5 **Anti-P28 does not inhibit binding or uptake of** ***E. chaffeensis*** **by THP-1 cells, related to** **Fig. 1D-F** **.** (A) Relative radioactivity representing numbers of *E. chaffeensis* bound to THP-1 cells. Host cell-free radiolabeled *E. chaffeensis* preincubated with Fab fragment of rabbit anti-P28 IgG or pre-immune rabbit IgG were incubated with THP-1 cells for 2 h at 4°C. Unbound *E. chaffeensis* was washed away, and radioactivity of bound *E. chaffeensis* was measured. (B) Relative radioactivity representing numbers of *E. chaffeensis* internalized into THP-1 cells. Host cell-free radiolabeled *E. chaffeensis* preincubated with Fab fragment of rabbit anti-P28 IgG or pre-immune rabbit IgG was incubated with THP-1 cells for 3 h at 37°C. Bound un-internalized *E. chaffeensis* was removed by pronase E treatment, radioactivity of internalized *E. chaffeensis* measured. Data represent the mean and standard deviation of triplicate samples and are representative of two independent experiments.
(TIF)

Figure S6 **Anti-EtpE-N is not effective in neutralizing** ***E. chaffeensis*** **infection** ***in vitro*** **and N-terminus of EtpE is less surface-accessible in live** ***E. chaffeensis*** **than its C-terminus, related to** **Fig. 1** **.** (A) Infection of RF/6A cells with *E. chaffeensis*. *E. chaffeensis* was pretreated with anti-EtpE-N or preimmune rabbit serum and used to infect RF/6A cells; cells were harvested at 48 h pi. qPCR for *E. chaffeensis* 16S rDNA was normalized with monkey G3PDH DNA. Data represent the mean and standard deviation of triplicate samples and are representative of three independent experiments. *Significantly different (*P*<0.05). (B) Immunofluorescence labeling of live host cell-free *E. chaffeensis*. Unfixed *E. chaffeensis* was first incubated with anti-EtpE-C, EtpE-N, or P28 (ECHP28); then fixed and labeled with AF555–conjugated secondary antibodies. Scale bar, 10 µm.
(TIF)

Figure S7 **MDC blocks entry of** ***E. chaffeensis*** **into non-phagocytic RF/6A cells, related to** **Fig. 4E** **.** Immunofluorescence labeling of *E. chaffeensis* incubated with RF/6A cells pre-treated with MDC or DMSO control. At 3 h pi, cells were treated with trypsin to remove un-internalized *E. chaffeensis* and then labeled with anti-P28. Scale bar, 10 µm. Bar graph shows quantitation by scoring *E. chaffeensis* (*Ech*) in 100 cells (right panel). Data represent the mean and standard deviation of triplicate samples and are representative of three independent experiments. * Significantly different (*P*<0.05).
(TIF)

Figure S8 **rEtpE-C-coated beads recruit DNase X to the areas of binding, related to** **Fig. 6** **.** rEtpE-C-coated or non-coated latex beads were incubated with canine primary macrophages derived from peripheral blood monocytes (A) or DH82 cells (B) at 37°C for 30 min, and labeled with anti-DNase X without permeabilization. rEtpE-C-coated beads recruited surface exposed DNase X to their sites of binding and clustered, whereas non-coated beads did not colocalize with DNase X on the cell surface. A single *z*-plane, of an optical section thickness of 0.4-µm, at cell surface by deconvolution microscopy was shown. Scale bar, 5 µm.
(TIF)

Figure S9 **Binding of rEtpE-C-coated beads is dependent on DNase X, related to** **Fig. 6C and D** **.** (A) Fluorescence and DIC merged images of rEtpE-C-coated, rECH0825-coated and non-coated beads incubated with BMDMs from wild-type and DNase X^−/−^ mice. Beads were incubated with cells for 30 min at 4°C followed by rigorous washing with PBS to remove unbound or loosely-adherent beads. Scale bar, 5 µm. (B) Numbers of internalized rEtpE-C-coated beads/cell of similar experiment as (A), relative to the number of rECH0825-coated beads bound to wild-type BMDM set as 100. Data represent the mean and standard deviation of triplicate samples and are representative of three independent experiments. *Significantly different (*P*<0.05).
(TIF)

Movie S1 **DNase X is recruited to the sites of binding of rEtpE-C-coated beads on cell surface, related to** **Fig. 5E** **.** Time-lapse video of rEtpE-C-coated latex beads binding to RF/6A cells ectopically expressing DNaseX-GFP. Coated beads were incubated with DNaseX-GFP-expressing RF/6A cells at 4°C, and the time 0 min was set upon raising the temperature to 37°C. The DNase X-GFP patchy signal is initially ∼3 µm away from the beads and redistributes towards the beads, eventually merging with the beads at around 5:47 min. A single *z*-plane, of an optical section thickness of 0.4-µm, at cell surface by deconvolution microscopy was shown. Scale bar, 30 µm.
(MOV)

Movie S2 **rEtpE-C-coated beads cluster with surface DNase X in phagocytic cells, related for** **Figs. 6A and 6B** **.** A 3-D view reconstructed from serial *z*-stack images (for a combined *z*-section width of 7.2 µm) of human primary macrophages derived from peripheral blood monocytes incubated with rEtpE-C-coated beads at 30 min pi, labeled with α-DNase X without permeabilization. rEtpE-C-coated beads cluster and colocalize with DNase X on the cell surface.
(MOV)

Movie S3 **Non-coated beads do not cluster with surface DNase X in phagocytic cells, related for** **Figs. 6A and 6B** **.** A 3-D view reconstructed from serial *z*-stack images (for a combined *z*-section width of 7.2 µm) of human primary macrophages derived from peripheral blood monocytes incubated with non-coated beads at 30 min pi, labeled with α-DNase X without permeabilization. Non-coated beads are randomly dispersed and not associated with DNase X, unlike the rEtpE-C-coated beads.
(MOV)
