# Supplementary figures and images for: Ehrlichia chaffeensis Uses Its Surface Protein EtpE to Bind GPI-Anchored Protein DNase X and Trigger Entry into Mammalian Cells
Source: PLoS Pathog. 2013 Oct 3;9(10):e1003666. doi: 10.1371/journal.ppat.1003666 (PMC3789761; doi:10.1371/journal.ppat.1003666)

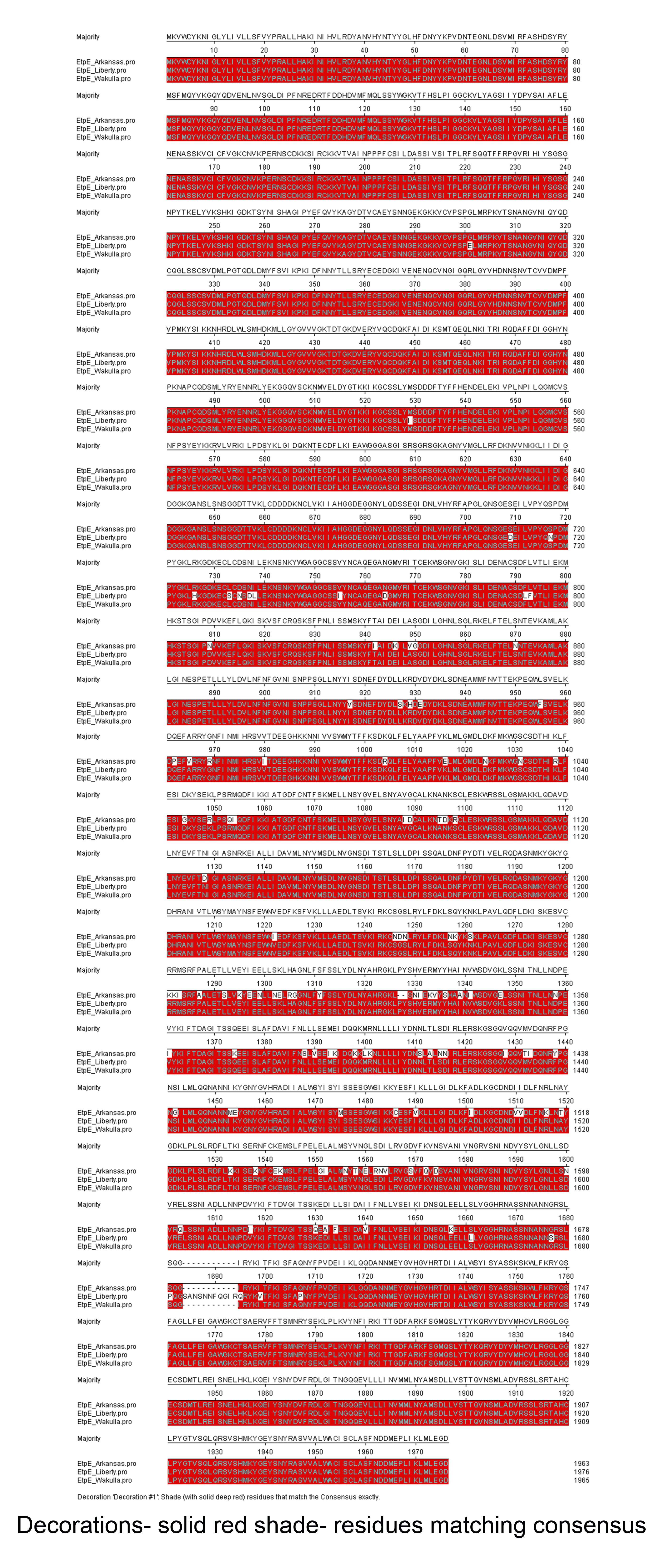

Supplement: Figure S1 — Alignment of Amino acid sequence of EtpE orthologs among three sequenced Ehrlichia chaffeensis strains, related to Fig. 1 . E. chaffeensis EtpEs of Arkansas, Wakulla, and Liberty (GenBank accession no. YP_507823.1, DQ915979.1 and DQ924562.1, respectively), were aligned by Clustal W using MegAlign. The red shade represents residues that match the consensus. (TIF) [file ppat.1003666.s001.tif]

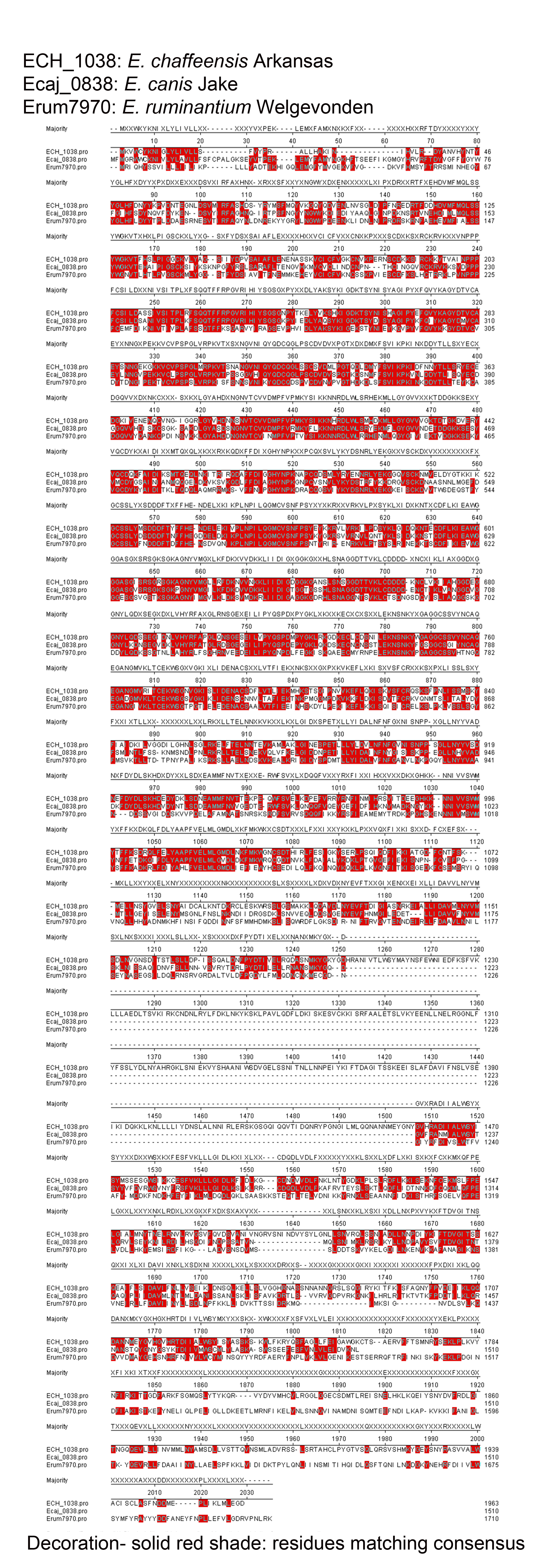

Supplement: Figure S2 — Alignment of Amino acid sequence of EtpE orthologs among three sequenced Ehrlichia species, related to Fig. 1 . E. chaffeensis Arkansas EtpE, E. canis Jake Ecaj_0838 and E. ruminantium Welgevonden Erum7970 (GenBank accession no. YP_507823.1, AAZ68869.1 and YP_180660.1, respectively), were aligned by Clustal W using MegAlign. The red shade represents residues that match the consensus. (TIF) [file ppat.1003666.s002.tif]

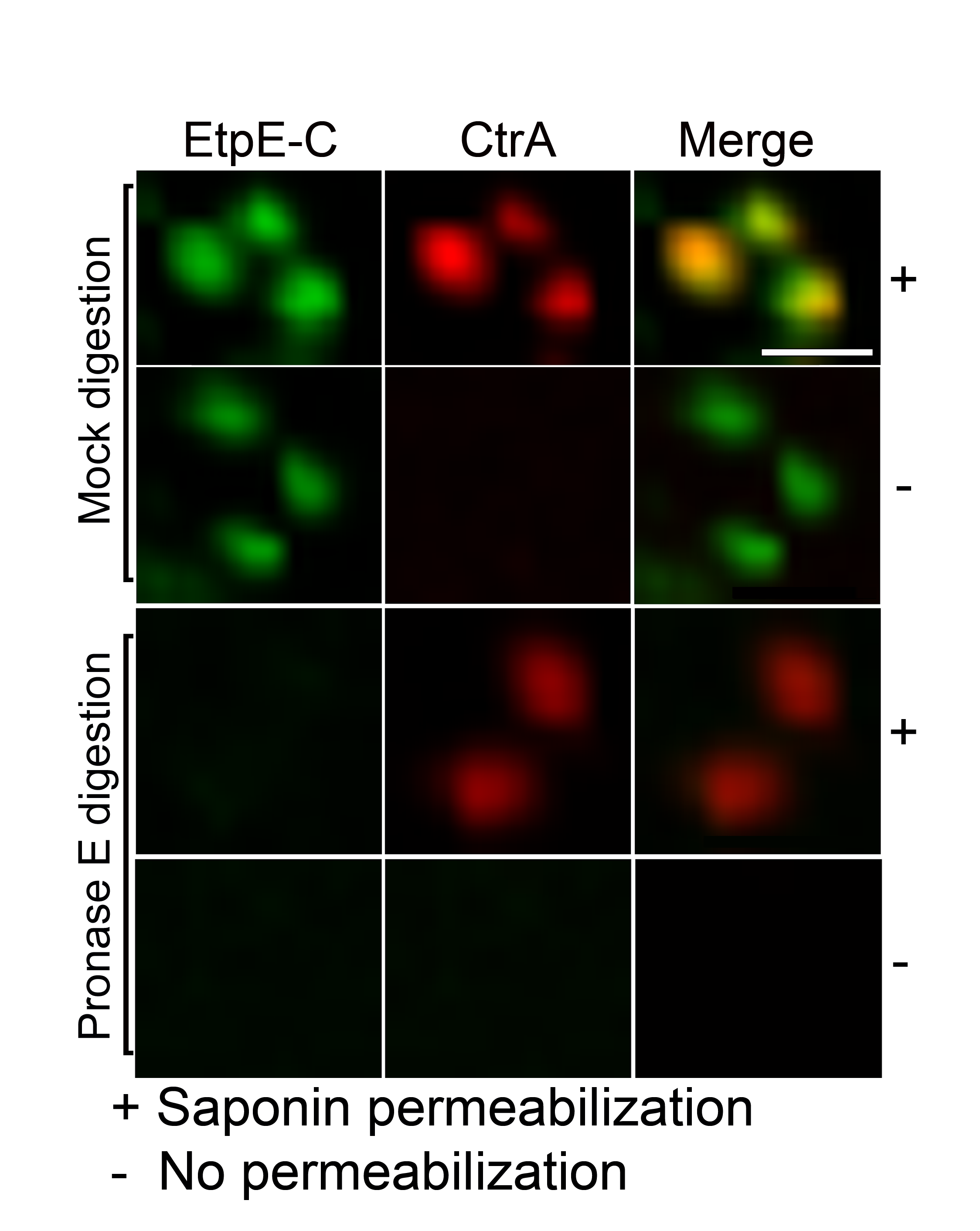

Supplement: Figure S3 — EtpE is bacterial surface exposed, related to Fig. 1C . Immunofluorescence image showing host cell-free E. chaffeensis that was either treated with pronase E or PBS control. Cells were processed for double immunostaining with anti-EtpE-C and anti-CtrA with or without saponin permeabilization as described to distinguish extracellular and internalized bacteria. When bacteria were treated with pronase E, the surface immunofluorescence staining of EtpE was abolished completely, but not that of the internal control CtrA. Scale bar, 1 µm. (TIF) [file ppat.1003666.s003.tif]

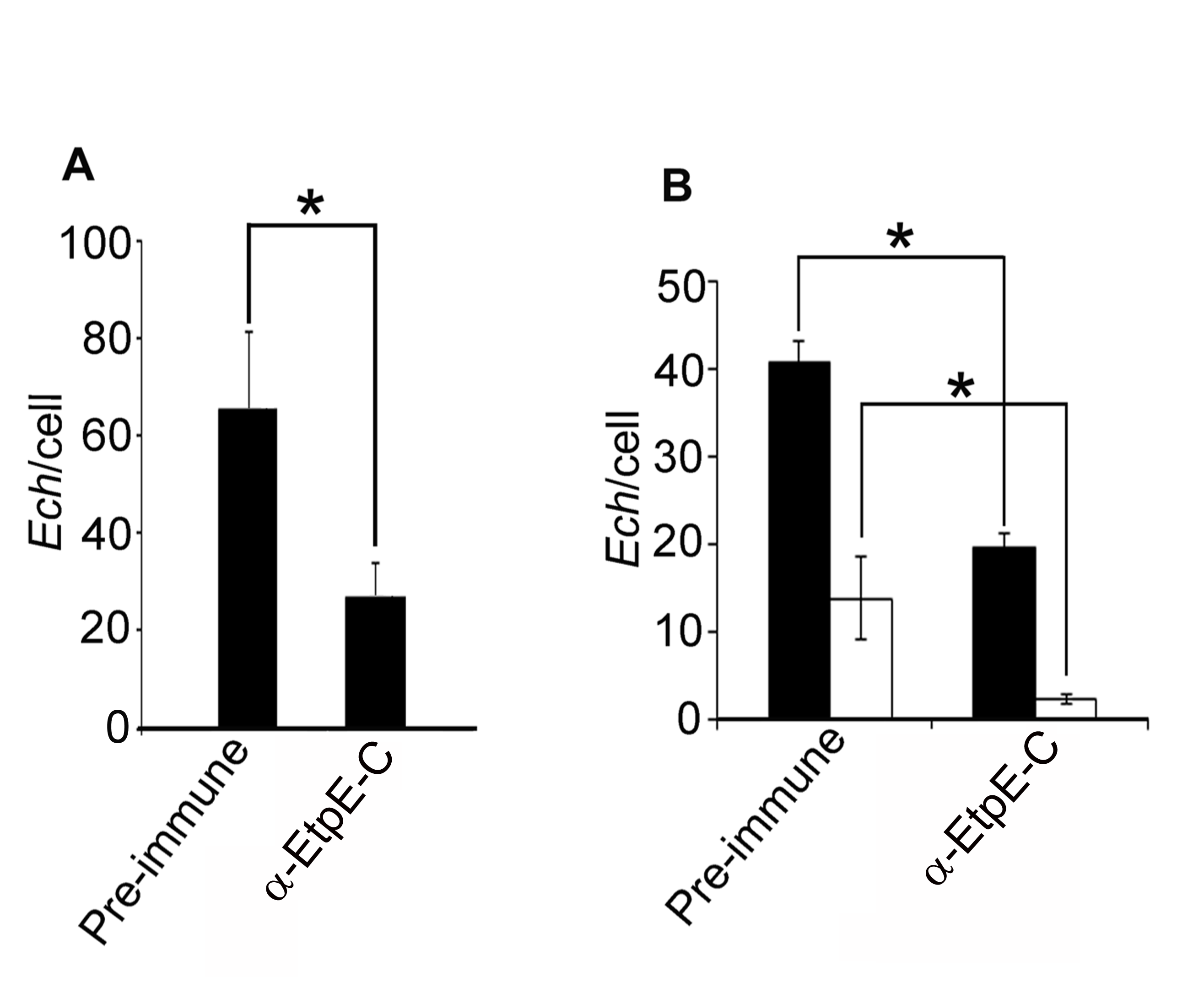

Supplement: Figure S4 — Anti-EtpE-C neutralizes E. chaffeensis binding and entry into THP-1 cells, related to Fig. 1D-F . (A) Numbers of E. chaffeensis (Ech) bound to THP-1 cells at 30 min pi. E. chaffeensis was pretreated with anti-EtpE-C or preimmune mouse serum and incubated with THP-1 cells for 30 min. Unbound E. chaffeensis was washed away, cells were fixed with PFA and E. chaffeensis was labeled with anti-P28 without permeabilization. E. chaffeensis in 100 cells was scored. (B) Numbers of E. chaffeensis internalized into THP-1 cells at 2 h pi. Purified host cell-free E. chaffeensis was pretreated with anti-rEtpE-C or preimmune mouse serum and incubated with THP-1 cells for 2 h. To distinguish intracellular from bound E. chaffeensis, unbound E. chaffeensis was washed away, and cells were processed for two rounds of immunostaining with anti-P28: first without permeabilization to detect bound but not internalized E. chaffeensis (AF555–conjugated secondary antibody), and another round with saponin permeabilization to detect total E. chaffeensis, i.e., bound plus internalized (AF488–conjugated secondary antibody). The black bar represents total E. chaffeensis, and the white bar represents internalized E. chaffeensis (total minus bound). E. chaffeensis in 100 cells was scored. qPCR for E. chaffeensis 16S rDNA was normalized with human G3PDH DNA. Data represent the mean and standard deviation of triplicate samples and are representative of three independent experiments. *Significantly different (P<0.05). (TIF) [file ppat.1003666.s004.tif]

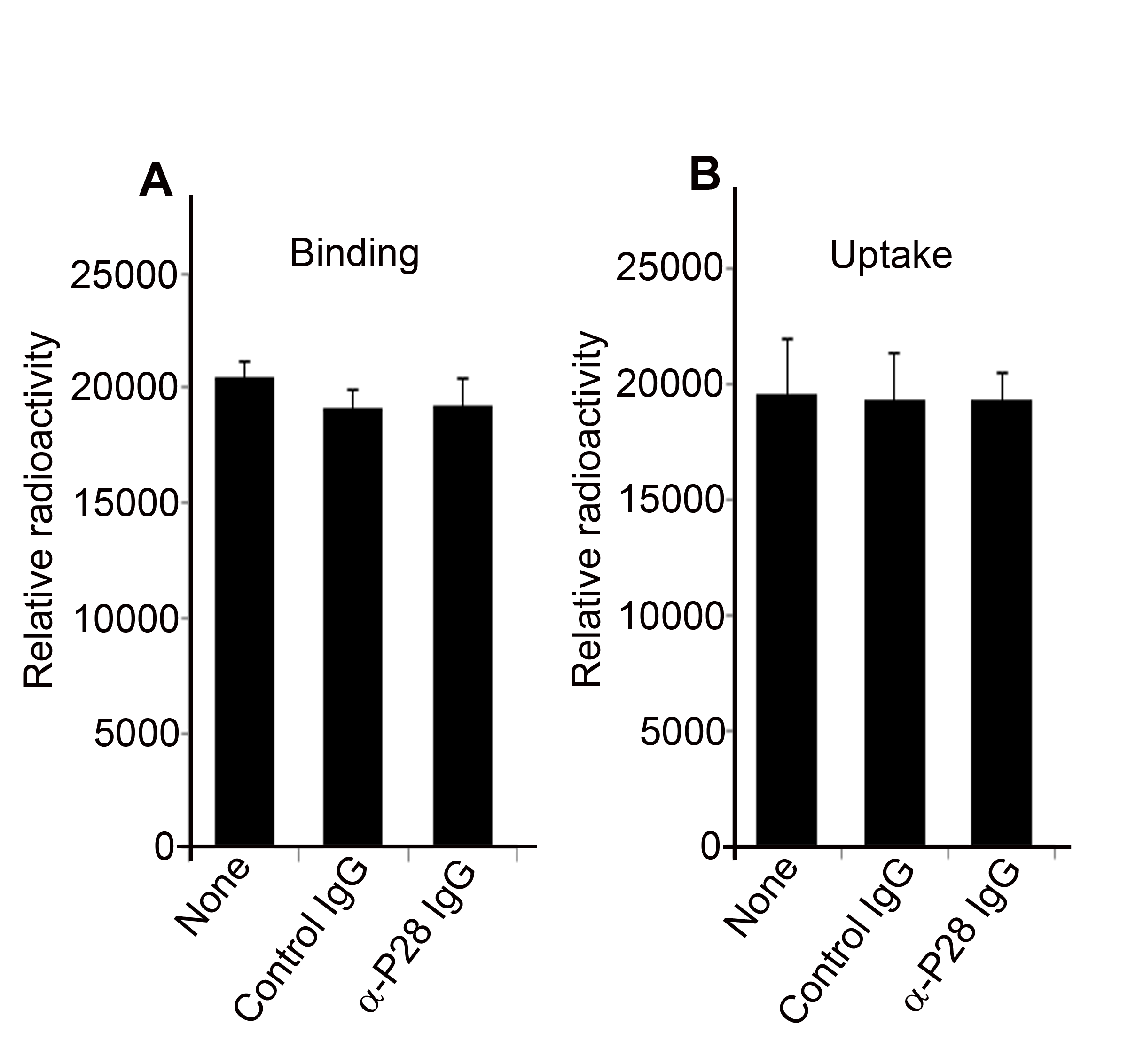

Supplement: Figure S5 — Anti-P28 does not inhibit binding or uptake of E. chaffeensis by THP-1 cells, related to Fig. 1D-F . (A) Relative radioactivity representing numbers of E. chaffeensis bound to THP-1 cells. Host cell-free radiolabeled E. chaffeensis preincubated with Fab fragment of rabbit anti-P28 IgG or pre-immune rabbit IgG were incubated with THP-1 cells for 2 h at 4°C. Unbound E. chaffeensis was washed away, and radioactivity of bound E. chaffeensis was measured. (B) Relative radioactivity representing numbers of E. chaffeensis internalized into THP-1 cells. Host cell-free radiolabeled E. chaffeensis preincubated with Fab fragment of rabbit anti-P28 IgG or pre-immune rabbit IgG was incubated with THP-1 cells for 3 h at 37°C. Bound un-internalized E. chaffeensis was removed by pronase E treatment, radioactivity of internalized E. chaffeensis measured. Data represent the mean and standard deviation of triplicate samples and are representative of two independent experiments. (TIF) [file ppat.1003666.s005.tif]

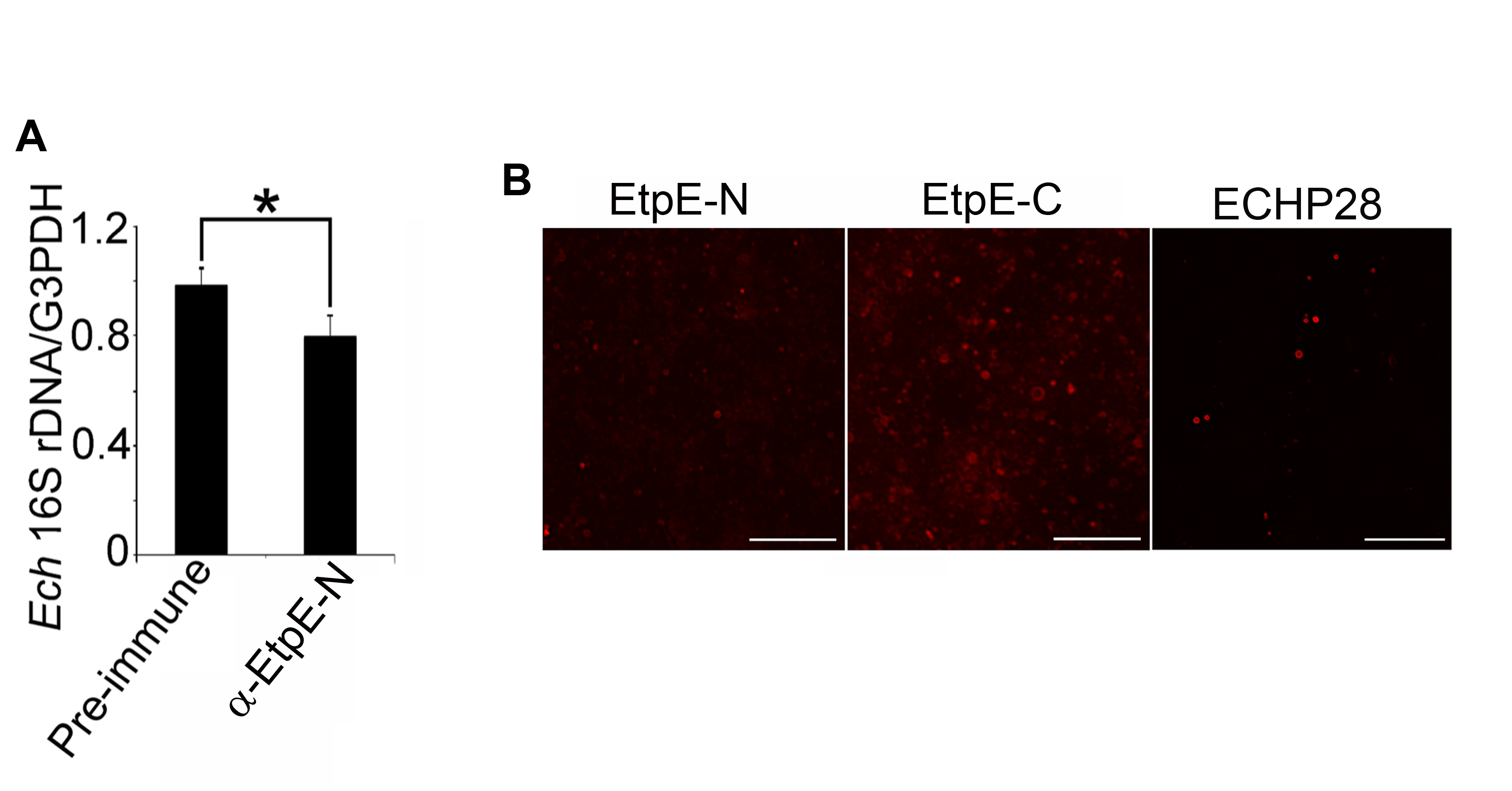

Supplement: Figure S6 — Anti-EtpE-N is not effective in neutralizing E. chaffeensis infection in vitro and N-terminus of EtpE is less surface-accessible in live E. chaffeensis than its C-terminus, related to Fig. 1 . (A) Infection of RF/6A cells with E. chaffeensis. E. chaffeensis was pretreated with anti-EtpE-N or preimmune rabbit serum and used to infect RF/6A cells; cells were harvested at 48 h pi. qPCR for E. chaffeensis 16S rDNA was normalized with monkey G3PDH DNA. Data represent the mean and standard deviation of triplicate samples and are representative of three independent experiments. *Significantly different (P<0.05). (B) Immunofluorescence labeling of live host cell-free E. chaffeensis. Unfixed E. chaffeensis was first incubated with anti-EtpE-C, EtpE-N, or P28 (ECHP28); then fixed and labeled with AF555–conjugated secondary antibodies. Scale bar, 10 µm. (TIF) [file ppat.1003666.s006.tif]

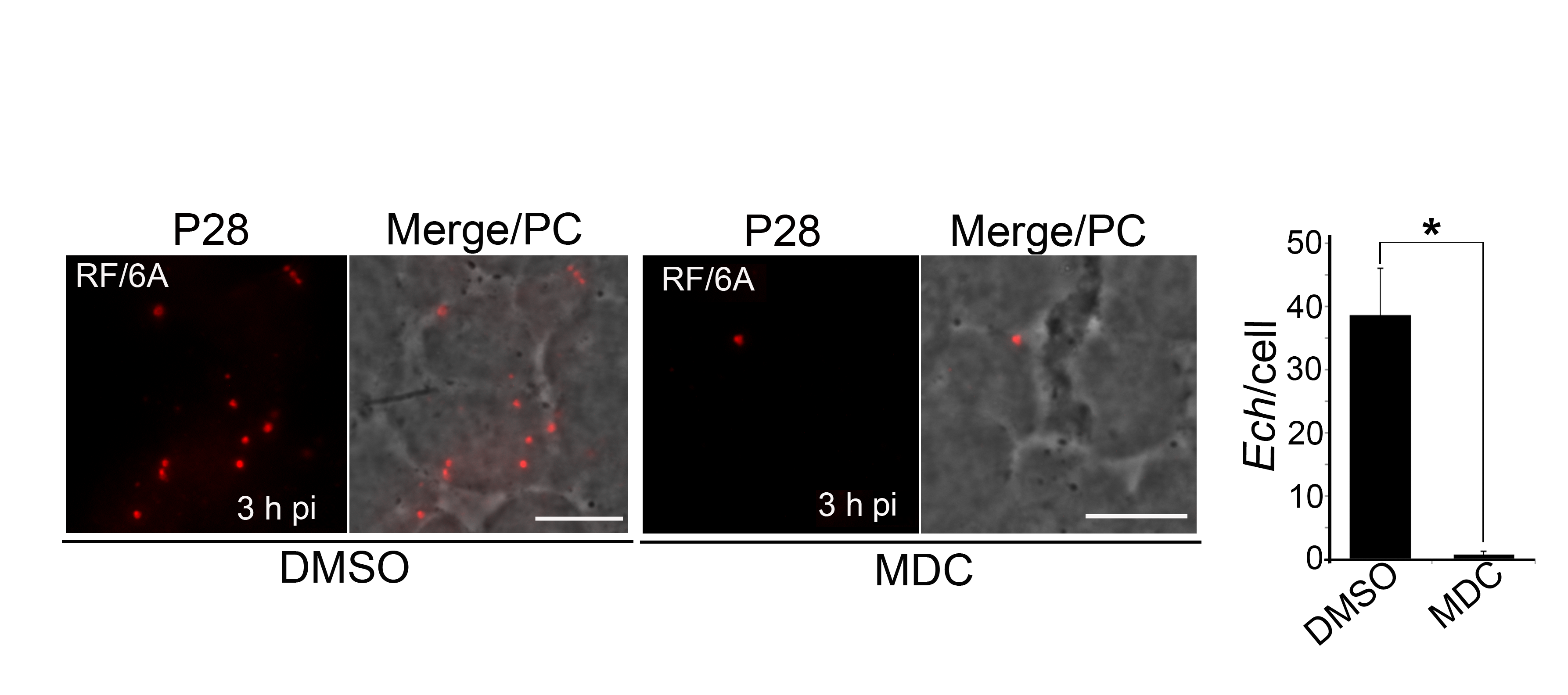

Supplement: Figure S7 — MDC blocks entry of E. chaffeensis into non-phagocytic RF/6A cells, related to Fig. 4E . Immunofluorescence labeling of E. chaffeensis incubated with RF/6A cells pre-treated with MDC or DMSO control. At 3 h pi, cells were treated with trypsin to remove un-internalized E. chaffeensis and then labeled with anti-P28. Scale bar, 10 µm. Bar graph shows quantitation by scoring E. chaffeensis (Ech) in 100 cells (right panel). Data represent the mean and standard deviation of triplicate samples and are representative of three independent experiments. * Significantly different (P<0.05). (TIF) [file ppat.1003666.s007.tif]

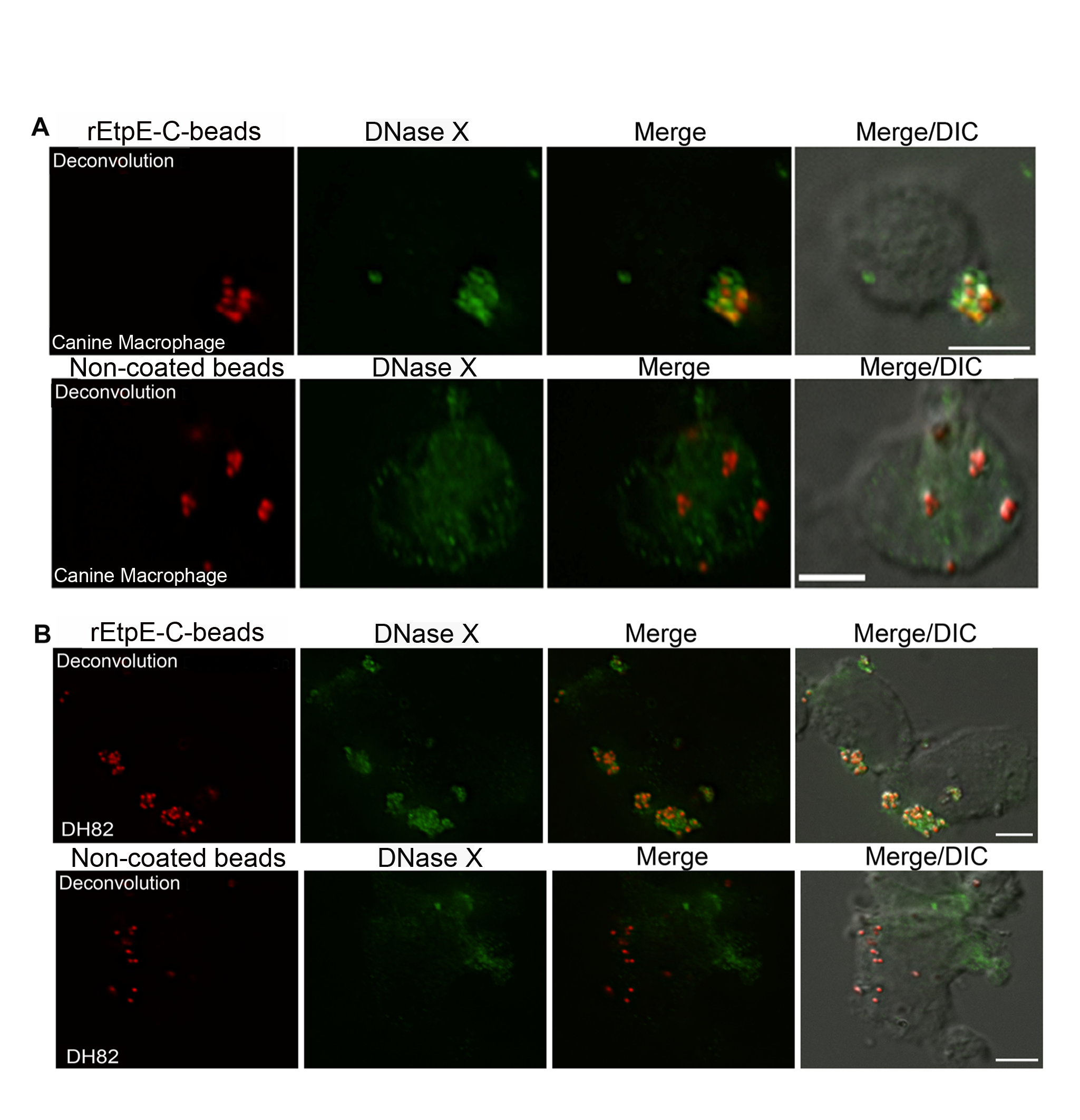

Supplement: Figure S8 — rEtpE-C-coated beads recruit DNase X to the areas of binding, related to Fig. 6 . rEtpE-C-coated or non-coated latex beads were incubated with canine primary macrophages derived from peripheral blood monocytes (A) or DH82 cells (B) at 37°C for 30 min, and labeled with anti-DNase X without permeabilization. rEtpE-C-coated beads recruited surface exposed DNase X to their sites of binding and clustered, whereas non-coated beads did not colocalize with DNase X on the cell surface. A single z-plane, of an optical section thickness of 0.4-µm, at cell surface by deconvolution microscopy was shown. Scale bar, 5 µm. (TIF) [file ppat.1003666.s008.tif]

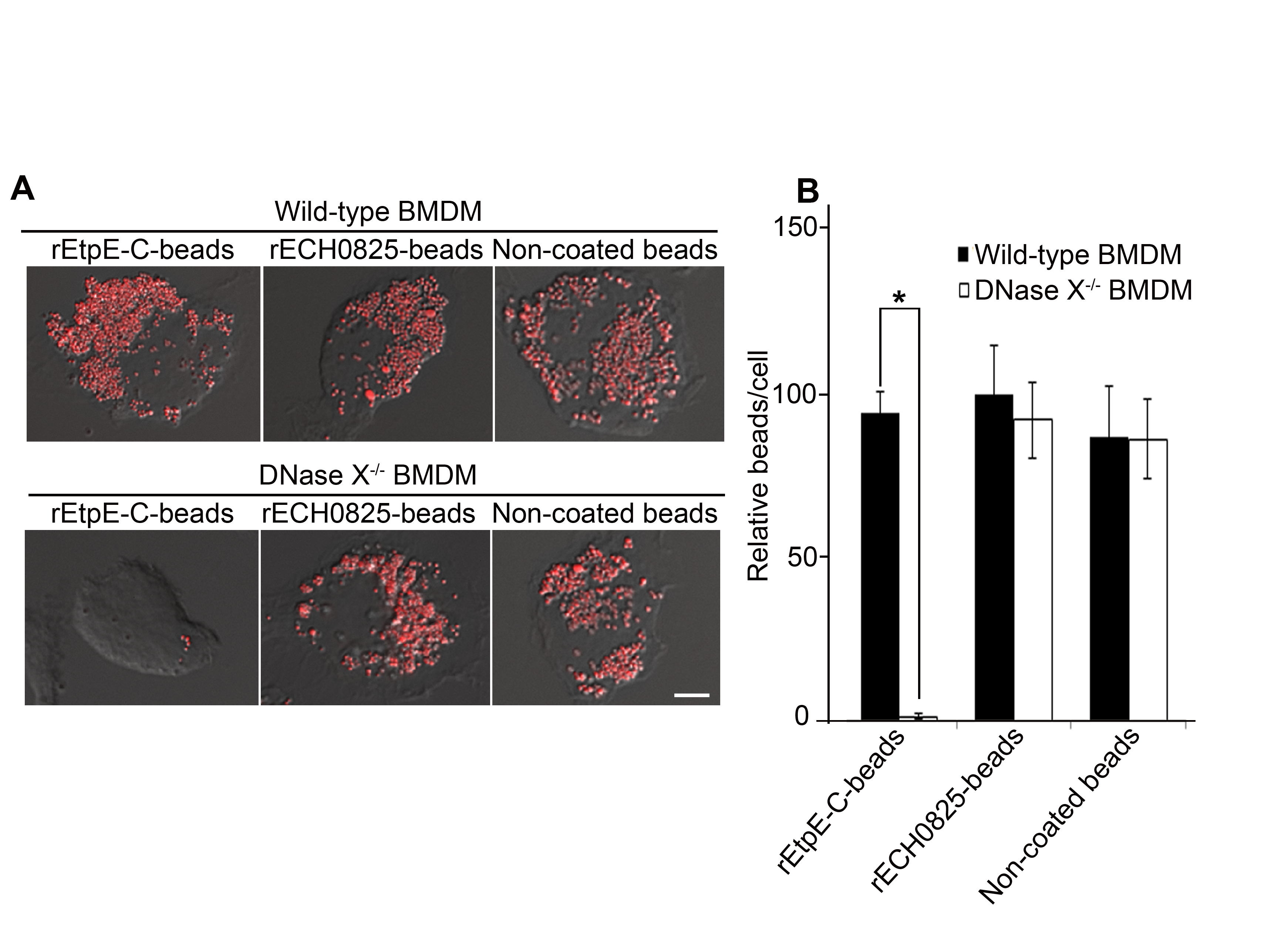

Supplement: Figure S9 — Binding of rEtpE-C-coated beads is dependent on DNase X, related to Fig. 6C and D . (A) Fluorescence and DIC merged images of rEtpE-C-coated, rECH0825-coated and non-coated beads incubated with BMDMs from wild-type and DNase X−/− mice. Beads were incubated with cells for 30 min at 4°C followed by rigorous washing with PBS to remove unbound or loosely-adherent beads. Scale bar, 5 µm. (B) Numbers of internalized rEtpE-C-coated beads/cell of similar experiment as (A), relative to the number of rECH0825-coated beads bound to wild-type BMDM set as 100. Data represent the mean and standard deviation of triplicate samples and are representative of three independent experiments. *Significantly different (P<0.05). (TIF) [file ppat.1003666.s009.tif]
